# Effect of audit and feedback with peer review on general practitioners’ prescribing and test ordering performance: a cluster-randomized controlled trial

**DOI:** 10.1186/s12875-017-0605-5

**Published:** 2017-04-13

**Authors:** J. Trietsch, B. van Steenkiste, R. Grol, B. Winkens, H. Ulenkate, J. Metsemakers, T. van der Weijden

**Affiliations:** 1 School for Public Health and Primary Care (CAPHRI), Department of Family Medicine, Maastricht University, PO Box 616, , 6200 MD Maastricht, The Netherlands; 2 IQ Healthcare, Radboud University Nijmegen, PO Box 9101 (144), , 6500HB Nijmegen, The Netherlands; 3 School for Public Health and Primary Care (CAPHRI), Department of Methodology and Statistics, Maastricht University, PO Box 616, , 6200 MD Maastricht, The Netherlands; 4 Department of Clinical Chemistry, ZorgSaam Hospital, Wielingenlaan 2, 4535 PA Terneuzen, The Netherlands

**Keywords:** Physician’s practice patterns, Education, Medical, Continuing/methods, Clinical audit, Clinical evaluation, Physician prescribing pattern

## Abstract

**Background:**

Much research worldwide is focussed on cost containment and better adherence to guidelines in healthcare. The research focussing on professional behaviour is often performed in a well-controlled research setting. In this study a large-scale implementation of a peer review strategy was tested on both test ordering and prescribing behaviour in primary care in the normal quality improvement setting.

**Methods:**

We planned a cluster-RCT in existing local quality improvement collaboratives (LQICs) in primary care. The study ran from January 2008 to January 2011. LQICs were randomly assigned to one of two trial arms, with each arm receiving the same intervention of audit and feedback combined with peer review. Both arms were offered five different clinical topics and acted as blind controls for the other arm. The differences in test ordering rates and prescribing rates between both arms were analysed in an intention-to-treat pre-post analysis and a per-protocol analysis.

**Results:**

Twenty-one LQIC groups, including 197 GPs working in 88 practices, entered the trial. The intention-to-treat analysis did not show a difference in the changes in test ordering or prescribing performance between intervention and control groups. The per-protocol analysis showed positive results for half of the clinical topics. The increase in total tests ordered was 3% in the intervention arm and 15% in the control arm. For prescribing the increase in prescriptions was 20% in the intervention arm and 66% in the control group. It was observed that the groups with the highest baseline test ordering and prescription volumes showed the largest improvements.

**Conclusions:**

Our study shows that the results from earlier work could not be confirmed by our attempt to implement the strategy in the field. We did not see a decrease in the volumes of tests ordered or of the drugs prescribed but were able to show a lesser increase instead. Implementing the peer review with audit and feedback proved to be not feasible in primary care in the Netherlands.

**Trial Registration:**

This trial was registered at the Dutch trial register under number ISRCTN40008171 on August 7^th^ 2007.

**Electronic supplementary material:**

The online version of this article (doi:10.1186/s12875-017-0605-5) contains supplementary material, which is available to authorized users.

## Background

Spiralling healthcare costs are a major concern for policymakers worldwide. Overuse, underuse and misuse of healthcare are estimated to be responsible for 30% of the total spending on healthcare annually. It has been estimated that 7% of the wasted healthcare spending in the US is due to overtreatment, including test ordering and prescribing [1]. In the years 2004–2011, the average annual growth in the number of prescriptions in the Netherlands was 5.7%; in fact, the growth of the national income of the Netherlands has been smaller than the growth of the healthcare budget year after year [2, 3]. If nothing is done to reduce the growth in healthcare spending, it is feared that Western countries will not be able to pay the healthcare bill in the long term. Therefore, physicians are being targeted by policymakers to contribute on reducing waste in healthcare, and are encouraged to alter their habits.

An unsolved problem with changing professional behaviour is the lack of a clear and solid benchmark for the desired behaviour [4, 5]. This can be overcome by using practice variations as a proxy for quality of care. A certain degree of practice variation is clearly warranted, given the unique profiles of individual patients and practice populations. However, when practice variation is caused by underuse or overuse of care, this results in unwarranted variation and thus inappropriate care [6]. In the Netherlands, general practitioners (GPs) now have access to over 100 evidence-based clinical practice guidelines. These guidelines have been developed by the Dutch College of General Practitioners (NHG) with the aim of reducing unwarranted practice variation and improving the quality of care provided. Although the general adherence to these guidelines seems quite reasonable, viz. approximately 70%, there is considerable practice variation in test ordering and prescribing, indicating room for improvement in poorly performing practices [7–12].

In local quality improvement collaboratives (LQICs), general practitioners meet on a regular basis to discuss current issues and gain new insights concerning test ordering and prescribing behaviour. Healthcare organisations and governments promote these meetings as a means to implement guidelines. LQICs are widely implemented in primary care, mainly in Europe and, to a lesser degree, in North America. Local pharmacists are members of these groups and are well respected for their input and knowledge.

These LQICs are an attractive target for interventions aimed at changing professional behaviour both effectively and efficaciously [13–20]. In a robust trial on three clinical topics, Verstappen et al. showed the beneficial effects of a multifaceted strategy involving audit and feedback with peer review in LQICs on test ordering behaviour. They found a reduction in the volumes of tests ordered ranging from 8 to 12% for the various clinical topics [21, 22]. Lagerlov et al. showed that individual feedback embedded in local peer group discussions improved appropriate treatment of asthma patients by 21% and urinary tract infections by 108%, compared to baseline values [23]. There is also evidence suggesting that the mere provision of information on test fees when presented at the time of the order entry reduces the volumes of tests ordered [24].

Most of this evidence, however, stems from trials focussing on a single or limited number of clinical topics, and involving a strong influence of the researcher on the participants, e.g. as moderator during sessions. Moreover, in the Verstappen trial, the included groups were selected by the researcher and can be regarded as innovator groups. We wanted to build on the experiences from the work by Verstappen et al. and undertake a large-scale implementation of the strategy in a pragmatic trial with much room for the LQICs to adapt the strategy to their own needs and without any researchers being present embedded within the existing network of LQICs.

We hypothesized that our intervention would reduce inappropriate testing and prescribing behaviour. Our research question was therefore: What is the effect of audit and feedback with peer review on general practitioners’ prescribing and test ordering performance?

We also report the sum scores of volumes of tests and prescriptions in a per-protocol analysis. This analysis was not planned in the study protocol [16], but we decided to add it as the process evaluation of the study revealed that the uptake of the strategy was much lower than expected [25].

## Methods

### Design

We conducted a two-arm cluster-randomised trial with the LQIC as the unit of randomisation and with central allocation. Core elements of the intervention are audit and comparative feedback on test ordering and prescribing volumes, dissemination of guidelines and peer review in quality improvement collaboratives moderated by local opinion leaders [16].

The intervention started in January 2008 and was completed as planned at the end of December 2010. We measured baseline performance during the six months before the intervention, and follow-up performance during the six months after the intervention. The design of this intervention is described in more detail in the trial protocol [16].

### Setting and Participants

Recruitment was restricted to the south of the Netherlands, because of our access to prescribing data of GPs working in this area. First, the regional health officers or key laboratory specialists for all 24 primary care diagnostic facilities in the south of the Netherlands were identified and recruited by the first author. They were trained in a three hour session in their region by the researchers. Object of this training was to transfer knowledge on effectively discussing test ordering and prescribing behaviour, setting working agreements, on how to effectively moderate meetings and how to deal with questions on the validity of the feedback data or other aspects of the intervention. Also written and digital materials where made available to enable them to facilitate recruitment of LQIC groups. The routines of the LQICs were deliberately left unchanged as they represented normal quality improvement routines in primary care in the Netherlands. Only when test ordering was discussed a laboratory specialist from the diagnostic facility moderated the group discussion. The strategy was new to all participants in this trial.

### Intervention

**Fig. 1:**
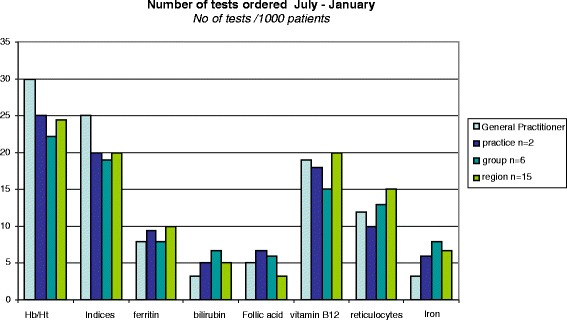
Example of the graphical comparative feedback (this image doesn’t reflect actual data)

In this trial with audit and feedback with peer review in LQICs we wanted to test the results on test ordering behaviour and prescribing behaviour of the strategy. Aggregated comparative feedback was provided on tests ordered or drugs prescribed in the period of six months before each meeting in which it was discussed. Feedback was sent to the moderator for that session (the local pharmacist or laboratory specialist). At the start of each meeting, each GP received feedback report on their own performance together with an outline of the recommendations from the guidelines, validated by clinical experts (Additional file 1: Appendix 1). The feedback was adjusted for practice size and compared with the aggregated results from their practice, their LQIC group and neighbouring groups (Fig. 1). To mimic the normal situation in self-directing LQIC groups, the groups in both arms were allowed to choose three clinical topics out of a set of five presented to them. The set of five topics differed between the two arms (Table 1). Each group planned two paired meetings for each topic, one on test ordering and one on prescribing making it a total of six meetings. Each meeting lasted between 90 and 120 min as was usual before depending on the intensity of the discussion. Groups were encouraged by the trained moderator (see under “setting and participants”) to establish working agreements to improve their performance, and to discuss barriers to change. The LQICs were allowed to adapt the format of the meeting to their own needs and routines, as long as peer review and working agreements were included. At the end of each meeting groups where asked to fill out a form stating what working agreements and goals were set.

**Table 1:** Sets of clinical topics and the number of meetings held for each topic

Clinical topic	Study arm	Number of meetings on test ordering	Number of meetings on prescribing	Desired direction of change	
				Tests	Drugs
Anaemia	A	9	9	↓	=
Dyslipedemia	A	2	2	↓	=
Prostate complaints	A	6	6	↓	↓
Rheumatic complaints	A	10	10	↓	=
UTI	A	2	2	↓	↓
Chlamydia trachomatis	B	8	8	↑	↑
Diabetes mellitus II	B	5	4	↓	=
Stomach complaints	B	6	6	↓	↓
Perimenopausal complaints	B	4	4	↓	↓
Thyroid dysfunction	B	6	6	↓	=
Total		58	57		

Feedback reports were generated from two main databases, one on diagnostic tests and one on prescriptions, with data originating from primary care diagnostic facilities and the two dominant insurance companies in the region. The databases contained data on the specific test or drug, the date it was ordered or prescribed, the practice in which the physician who had ordered or prescribed it worked, the date of birth of the patient, their gender and, in the case of prescriptions, the number of defined daily dosages (DDDs) that were prescribed. A more detailed description of the intervention is available in the previously published trial protocol [16].

### Data collection and main outcome measures

The primary outcome measures were the volumes of tests ordered and drugs prescribed per practice, per 1000 patients, per 6 months. Although data on a large number of diagnostic tests and prescriptions were available (Additional file 2: Appendix 2), only results on key tests and drugs for each clinical topic are reported in this paper. The identification of these key tests and drugs was based on consensus within the research group and one clinical expert on each topic before the intervention started.

### Sample size

We calculated that a total of 44 LQICs would be sufficient to detect a standardised effect size (Cohens d) of 0.5, with a significance level alpha of 0.05, a power of 0.9, an ICC of 0.1 and a mean group size of seven GPs. Anticipating a dropout rate of 10%, we would need to recruit 50 LQICs. [10, 16].

### Randomisation

Prior to the randomisation, the LQICs were stratified on their level of group performance, as assessed by a questionnaire resulting in four levels of group work, from ‘poor’ to ‘good’. The level of group performance may be a confounder for the ability to establish shared working agreements and for the quality of prescribing behaviour [26–29]. By stratifying on this, we ensured an equal distribution of these levels over the trial arms. An independent research assistant produced a computer-generated allocation list and allocated the LQICs to arm A or arm B, while the researcher was blinded to this process. Groups in both trial arms were exposed to the same intervention, but on different clinical topics. Each LQIC in one arm served as an unmatched control for the LQICs in the other arm [30, 31]. Groups were blinded to the clinical topics discussed in the other arm. The researcher was blinded until all data analyses had been completed.

### Data analysis

To analyse the intention-to-treat differences between the two arms we compared performances at the LQIC level for all key tests and drugs during the six months prior to the intervention with performances during the six months after completion of the intervention period. We analysed according to the intention-to-treat principle regardless whether a group had chosen a clinical topic or not. In addition, we performed a per-protocol before and after analysis to test for effects in the groups that had actually organised a meeting on a specific topic, with all other groups acting as controls in the analysis.

**Fig. 2:**
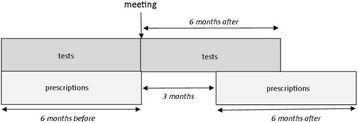
Graphical display of the periods defined for the baseline and follow-up measurement of tests ordered and drugs prescribed in the per protocol analysis

The time intervals for our per-protocol analyses were six months prior to each LQIC meeting, compared with 0–6 months after each LQIC meeting in the case of tests, and 3–9 months after each meeting in the case of prescribing (Fig. 2). By using this washout period we avoided contamination with long-term prescriptions.

We used a Chi-square test or *T*-test to check if the stratification of groups had led to an even distribution of the LQIC group performance levels and the characteristics of the participants over the trial arms and topics. The group effect (intervention versus control) on prescribing rate and test ordering rate after the intervention was assessed using a linear mixed model with the LQIC as a random effect to account for the clustering of practices within the LQIC. In addition, group (intervention or control), baseline value of the outcome measure (before the intervention) and interaction between the baseline and group were included as fixed factors. If the interaction term was not statistically significant, it was removed from the model, and only the overall group effect is presented. This method was used for both the intention-to-treat analysis and the per-protocol analysis.

If the interaction term was statistically significant, the overall group effect (obtained from the model without the interaction term) and the group effects for different baseline values, at the 10th and 90^th^ percentiles of the baseline variable, are presented to assess the effects at both ends of the spectrum. We expected that change would mostly be seen in GPs in the 90^th^ percentile, as this is a clear indication of overuse and marks a need to decrease test ordering or prescription volumes. Although it is not clear what the benchmark is for volumes of tests and prescriptions, we did not expect GPs at the other end of the spectrum—the 10th percentile—to clearly fail in terms of underuse of tests and prescriptions.

All data analyses were performed using IBM SPSS Statistics for Windows, Version 21.0 (Armonk, NY: IBM Corp). *P*-values ≤ 0.05 were considered statistically significant.

### Exclusion of data before analysis

At the time when the intervention was designed, the recommendations for dyslipidemia and type 2 diabetes mellitus were provided in two separate guidelines. However, at the start of the actual intervention, the guidelines on diabetes management and dyslipidemia treatment were merged into one new, multidisciplinary national guideline on cardiovascular risk management. This was directly followed by a massive government led intervention to transfer care for diabetics and cardiovascular risk patients from specialist care to GPs. Part of this transfer was the institution of a pay for performance model for these two topics together with the introduction of many outcome indicators being tracked by newly introduced and completely integrated software [32]. As part of this campaign much publicity was created in both the professional and public media. This caused substantial contamination of our intervention contrast, resulting in an inability to interpret the results on these clinical topics. Therefore, we chose to exclude the topics of dyslipidemia and type 2 diabetes mellitus from the analyses. The results on test ordering for the clinical topics of urinary tract infections (UTI) and stomach complaints could not be calculated either, due to insufficient data on test ordering from laboratories and diagnostic facilities to report these data (e.g., urine cultures and gastroscopies). Results on prescription rates for Chlamydia are not shown because it proved impossible to link the prescribed antibiotics reliably to this condition. This problem did not occur for UTI, as we confined the data to nitrofurantoin and trimethoprim, which are antibiotics that are only indicated or prescribed for UTI treatment in Dutch primary care.

## Results

### Participants

**Fig. 3:**
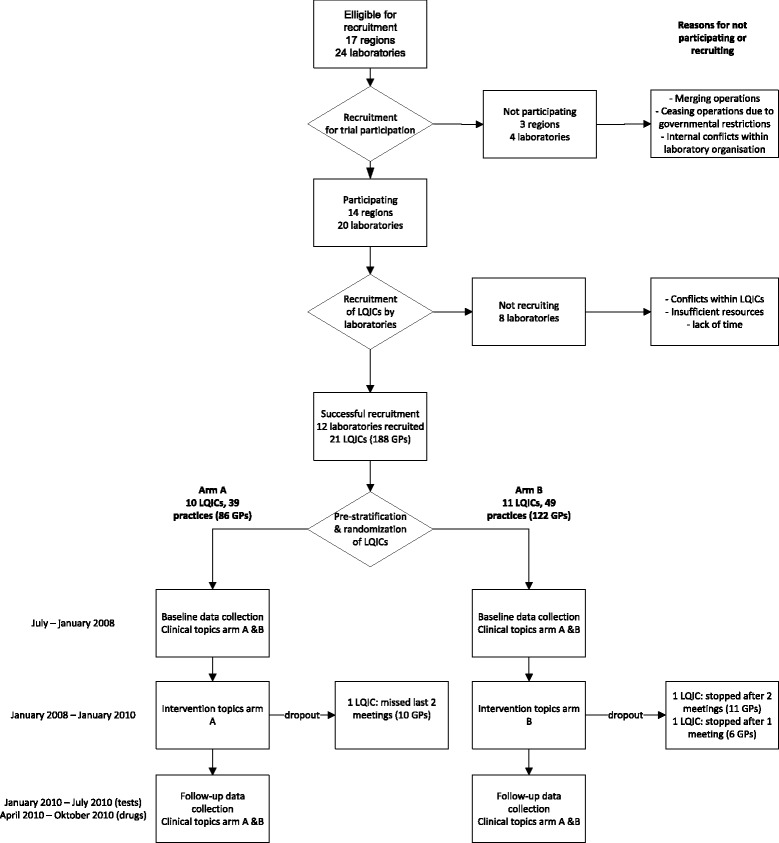
Flowchart of recruitment of laboratories, laboratory specialists or regional health officers and their recruitment of LQICs with the number of GPs in brackets

Out of the 24 primary care diagnostic facilities (laboratories) we approached, 12 actually managed to recruit 21 LQICs for the trial (Fig. 3). The other facilities did not manage to recruit due to various reasons, which is described in detail in the process evaluation. The 21 groups consisted of 197 GPs working in 88 practices, and 39 community pharmacists. Eight laboratory specialists participated in the groups when test ordering was being discussed. The characteristics of the participating groups and the GP members of these groups are described in Table 2.

**Table 2:** Characteristics of the participating GPs and groups

	Arm A	Arm B	*p*
Number of groups	10	11	0.40
Number of practices	39	49	0.45
Mean group size (GPs per group)	8.1 (0.64^a^)	10.55 (1.70^a^)	0.20
Mean group level	2.70 (0.26^a^)	2.82 (0.33^a^)	0.78
No. of men (n total)	53 (81)	78 (116)	0.791
Mean age of GPs	47.9 (0.91^a^)	47.1 (0.77^a^)	0.538

^a^Standard deviation

### Results of the intention-to-treat analysis

The intention-to-treat before and after analyses on test ordering did not show any differences between the intervention and control groups, with wide confidence intervals, all including 0, and all with *p*-values well above 0.05 (Table 3). Interaction with the baseline values was present for rheumatic complaints. This showed a difference in the desired direction in number of tests ordered for the practices within the p90 range of test ordering only.

**Table 3:** Intention-to-treat analysis of changes in test ordering rates

	Intervention			Control						
Clinical topic	n	Mean before (sd)	Mean after (sd)	*n*	Mean before (sd)	Mean after (sd)	B^a^ (95% CI)	icc	Direction	*p*
Anaemia	39	177.8 (173.1)	208.4 (216.9)	49	208.1 (148.7)	247.3 (176.7)	15.3 (−53.7, 83.3)	0.31	↓	0.630
Rheumatic complaints	39	12.9 (20.1)	13.1 (23.0)	49	12.9 (16.2)	15.0 (27.5)	2.9 (−9.0, 14.9)	0.31	↓	0.581^b^
Prostate complaints	39	34.4 (28.0)	36.1 (26.2)	49	37.6 (27.9)	40.7 (31.0)	1.8 (−4.9, 8.6)	0.04	↓	0.575
Chlamydia infections	49	9.9 (15.4)	8.9 (19.5)	39	7.8 (9.6)	6.3 (10.0)	2.2 (−9.5, 13.9)	0.74	↑	0.701
Thyroid dysfunction	49	178.9 (139.0)	206.1 (155.4)	39	160.6 (158.3)	186.1 (151.7)	3.9 (−26.3, 34.11)	0.03	↓	0.788
Perimenopausal complaints	49	5.6 (6.2)	5.2 (6.0)	39	6.4 (7.6)	7.4 (8.5)	−1.4 (−5.0, 2.1)	0.29	↓	0.404

The desired direction of change is given in the column headed ‘Direction’. Numbers represent the number of tests prescribed per 6 months per 1000 patients

*n* = number of practices

^a^Adjusted difference between groups at end of intervention, corrected for baseline

^b^Statistically significant interaction

Rheumatic complaints

P 10: B (95% CI):−7.8 (−22.0, 6.5). p: 0.257

P 90: B (95% CI): 16.3 (1.7, 30.9). p: 0.031

The intention-to-treat analysis on drug prescriptions showed a difference in the desired direction for misoprostol only. Interaction with baseline values was present for misoprostol, the triple therapy for *Helicobacter pylori* eradication (PantoPac®), antithyroid preparation drugs and clonidine (Table 4), showing changes in the desired direction for misoprostol, antithyroid preparations and clonidine. This effect is not present for the prescription rates of triple therapy at either p10 and p90.

**Table 4:** Intention-to-treat analysis of changes in prescribing rates showing sum scores per clinical topic and scores per drug

	Intervention			Control						
Clinical topic	*n*	Mean before (sd)	Mean after (sd)	*n*	Mean before (sd)	Mean after (sd)	B^a^ (95% CI)	icc	Direction	*p*
Anaemia										
Iron preparations, oral	39	25.0 (20.7)	45.1 (40.0)	49	27.4 (28.4)	49.2 (55.7)	3.9 (−13.9, 21.5)	0.21	=	0.644
Urinary tract infections										
Antibiotics UTI	39	43.5 (31.6)	47.3 (36.5)	49	47.5 (38.7)	59.7 (48.7)	11.9 (−3.2, 27.0)	0.21	↓	0.113
Prostate complaints	39	59.1 (43.3)	107.1 (69.6)	49	66.5 (58.6)	127.5 (102.2)	20.0 (−20.9, 60.8)	0.38	↓	0.306
Alpha receptor blockers	39	49.6 (35.7)	86.6 (56.2)	49	55.0 (49.5)	103.3 (81.5)	18.5 (−15.3, 52.3)	0.42	↓	0.258
5 alpha reductase inhibitors	39	9.5 (9.5)	20.4 (18.6)	49	11.5 (11.2)	24.2 (24.7)	1.4 (−7.2, 10.0)	0.01	↓	0.735
Stomach complaints	49	329.7 (286.9)	612.0 (477.0)	39	282.6 (210.1)	491.8 (333.4)	106.7 (−70.7, 284.2)	0.63	↓	0.219
H2 antagonists	49	27.8 (26.4)	32.4 (30.0)	39	22.3 (20.5)	21.3 (16.4)	12.5 (−1.8, 26.8)	0.44	↑	0.081
Proton pump inhibitors	49	300.8 (262.9)	578.3 (452.3)	39	259.4 (192.3)	469.1 (322.1)	93.7 (−71.9, 259.4)	0.62	↓	0.246
Misoprostol	49	0.1 (0.4)	0.0 (0.2)	39	0.1 (0.4)	0.2 (0.5)	−0.1 (−0.2,−0.0)	0.00	↓	0.008 ^b^
Triple therapy (ATC A02BD04)	49	1.0 (1.7)	1.3 (2.0)	39	0.9 (1.3)	1.1 (1.7)	0.2 (−0.7, 1.2)	0.49	↓	0.593 ^b^
Thyroid dysfunction	49	81.3 (66.3)	137.2 (102.6)	39	71.4 (48.9)	107.1 (71.6)	21.2 (−14.1, 56.6)	0.42	=	0.217
Thyroid hormones	49	74.8 (60.8)	125.0 (92.6)	39	66.9 (47.4)	100.8 (70.0)	18.2 (−16.0, 52.4)	0.45	=	0.269
Antithyroid preparations	49	6.5 (8.6)	12.2 (17.8)	39	4.5 (4.4)	6.3 (7.3)	2.6 (−1.7, 7.0)	0.09	↑	0.218 ^b^
Perimenopausal complaints	49	56.4 (42.7)	56.8 (41.8)	39	46.3 (34.2)	46.0 (29.6)	6.6 (−12.0, 25.2)	0.56	↓	0.459
Oestrogens, oral	49	14.1 (13.6)	12.7 (12.1)	39	12.3 (11.8)	11.2 (10.4)	0.2 (−3.6, 4.1)	0.01	↓	0.889
Contraceptives (>50y)	49	3.6 (3.2)	6.4 (4.9)	39	3.2 (3.9)	5.9 (4.2)	1.1 (−1.9, 4.2)	0.34	↓	0.431
Hormone replacement therapy	49	34.8 (28.6)	34.7 (27.0)	39	28.3 (19.7)	26.3 (17.8)	5.8 (−6.5, 18.0)	0.70	↓	0.331
Clonidine	49	0.5 (1.8)	0.5 (2.0)	39	0.2 (0.5)	0.4 (2.0)	0.1 (−1.0, 1.2)	0.13	↓	0.908 ^b^
Tibolon	49	3.4 (4.1)	2.4 (2.7)	39	2.3 (2.8)	2.2 (3.4)	−0.3 (−1.4, 0.8)	0.00	↓	0.615

The desired direction of change is given in the column headed ‘Direction’. Numbers represent the number of DDDs prescribed per 6 months per 1000 patients

*n* = number of practices

^a^Adjusted difference between groups at end of intervention, corrected for baseline

^b^statistically significant interaction

Misoprostol

P 10: B (95% CI):−0.1 (−0.2,−0.0). p: 0.039

P 90: B (95% CI):−0.1 (−0.2,−0.0). p: 0.039

Triple therapy (ATC A02BD04)

P10: B (95% CI): 0.9 (−0.2, 1.9). p: 0.117

P90: B (95% CI):−0.4 (−1.6, 0.7). p: 0.413

Antithyroid preparations

P10: B (95% CI):−1.9 (−7.2, 3.4). p: 0.474

P90: B (95% CI): 12.3 (4.6, 19.9). p: 0.002

Clonidine

P10: B (95% CI): 0.6 (−0.4, 1.6). p: 0.215

P90: B (95% CI):−2.4 (−3.9,−1.0). p: < 0.001

### Results of the per-protocol results

Table 5 shows the results of the per-protocol analyses on test ordering volumes for all groups that covered a specific topic (intervention group) compared to all other groups (controls). We found a difference between both trial arms in the desired direction in test ordering only for thyroid dysfunction and perimenopausal complaints.

**Table 5:** Per-protocol analysis of change in test ordering rates for each topic

	Intervention			control						
Clinical topic	*n*	Mean before (SD)	Mean after (SD)	*n*	Mean before (SD)	Mean after (SD)	B^a^ (95% CI)	icc	Direction	*p*
Anaemia	35	176.8 (172.2)	209.4 (174.6)	53	199.6 (146.3)	232.5 (177.7)	−9.1 (−96.1, 77.9)	0.60	↓	0.829
Rheumatic complaints	39	13.3 (22.9)	11.6 (20.7)	49	12.9 (16.2)	14.9 (27.5)	3.2 (−8.1, 14.6)	0.55	↓	0.551^b^
Prostate complaints	26	34.7 (24.6)	30.4 (29.3)	62	39.1 (27.4)	41.8 (29.5)	6.9 (−0.7, 14.6)	0.07	↓	0.072
Chlamydia infections	30	11.4 (21.5)	14.7 (23.3)	58	7.4 (8.8)	5.8 (9.5)	−5.7 (−12.0, 0.5)	0.40	↑	0.069^b^
Thyroid dysfunction	29	204.8 (159.9)	191.3 (135.4)	59	163.3 (153.2)	191.7 (152.8)	36.6 (10.5, 62.7)	0.00	↓	0.007
Perimenopausal complaints	24	5.7 (5.5)	3.8 (3.5)	64	6.1 (6.7)	7.3 (8.0)	3.2 (0.1, 6.4)	0.10	↓	0.046^b^

The desired direction of change is given in the column headed ‘direction’. Numbers represent the number of tests prescribed per 6 months per 1000 patients

*n* = number of practices

^a^Adjusted difference between groups at end of intervention, corrected for baseline

^b^Statistically significant interaction

Rheumatic complaints

P10: B (95% CI):−3.0 (−13.9, 8.0). p: 0.560

P90: B (95% CI): 14.8 (2.1, 27.4). p: 0.025

Chlamydia infections

P10: B (95% CI):−2.3 (−9.0, 4.4) p: 0.482

P90: B (95% CI):−10.4 (−17.6,−3.3) p: 0.006

Perimenopausal complaints

P10: B (95% CI): 0.7 (−3.0, 4.5). p: 0.696

P90: B (95% CI): 6.6 (2.4, 10.7). p: 0.002

Testing for interaction with baseline measurements showed a difference in test ordering rates in the desired direction for those GP practices with a baseline test-ordering rate at or above p90 for chlamydia infections, rheumatic complaints and perimenopausal complaints.

Table 6 shows the results of the per-protocol analysis on prescribing performance. A difference in the overall volume of all prescribed drugs between intervention groups and their controls was observed for medication prescribed for prostate complaints (, stomach complaints and thyroid dysfunction. For each of the clinical topics we also analysed each Anatomical Therapeutic Chemical Classification System (ATC) group in that topic separately, as changes found for specific ATC groups could represent clinically relevant changes. These results are shown in more detail in Table 6. Testing for interaction with baseline measurements again showed a statistically significant interaction for several topics and specific ATC groups. All showed larger differences in prescribing rates before and after the intervention for the practices in the p90 range than for those in the p10 range (Table 6).

**Table 6:** Per-protocol analysis of change in prescribing rates, showing sum scores per clinical topic and drug

	Intervention			Control						
Clinical topic	*N*	Mean before (sd)	Mean after (sd)	*n*	Mean before (sd)	Mean after (sd)	B^a^ (95% CI)	icc	Direction	p
Anaemia										
Iron preparations. oral	35	29.5 (22.5)	38.7 (25.6)	53	26.6 (27.6)	46.9 (54.2)	18.9 (−3.9, 41.7)	0.43	=	0.098
Urinary tract infections										
Antibiotics UTI	8	40.8 (24.0)	44.5 (29.7)	80	46.5 (36.3)	55.3 (44.8)	6.3 (−21.1, 33.8)	0.33	↓	0.623
Prostate complaints	26	68.5 (47.0)	79.5 (63.0)	62	65.5 (53.6)	125.9 (91.9)	28.5 (6.5, 50.7)	0.27	↓	0.016
Alpha receptor blockers	26	57.5 (41.1)	66.5 (54.2)	62	54.0 (44.9)	101.5 (73.2)	44.2 (6.0, 82.3)	0.49	↓	0.027
5 alpha reductase inhibitors	26	11.0 (7.5)	13.0 (11.7)	62	11.5 (11.0)	24.5 (22.9)	11.1 (2.1, 20.2)	0.05	↓	0.020
Stomach complaints	28	455.1 (294.7)	565.2 (303.7)	60	275.7 (253.8)	509.1 (433.0)	61.1 (15.0, 107.2)	0.11	↓	0.014 ^b^
H2 antagonists	28	28.0 (22.4)	32.4 (26.8)	60	23.2 (22.35)	24.2 (22.6)	−2.4 (−21.5, 16.7)	0.68	↑	0.788
Proton pump inhibitors	28	425.9 (276.1)	531.5 (283.8)	60	251.6 (234.0)	483.7 (414.7)	254.8 (60.1, 449.5)	0.66	↓	0.014 ^b^
Misoprostol	28	0 (0)	0 (0)	60	0.1 (0.4)	0.1 (0.45)	0.1 (−0.1, 0.2)	0.01	↓	0.365
Triple therapy (ATC A02BD04)	28	1.2 (2.5)	1.4 (1.8)	60	0.9 (1.2)	1.1 (1.6)	0.1 (−0.7, 0.8)	0.20	↓	0.878^b^
Thyroid dysfunction	29	82.2 (45.9)	99.2 (57.5)	59	86.0 (63.1)	128.5 (94.6)	12.6 (0.7, 24.4)	0.08	=	0.040
Thyroid hormones	29	76.7 (43.5)	91.1 (52.2)	59	79.5 (58.8)	118.3 (87.8)	25.2 (2.1, 48.3)	0.19	=	0.035
Antithyroid preparations	29	5.5 (7.4)	8.2 (13.2)	59	6.5 (7.7)	10.1 (14.0)	0.2 (−4.2, 4.6)	0.11	↑	0.927
Perimenopausal complaints	24	59.4 (31.9)	56.5 (25.9)	64	51.2 (40.0)	51.3 (40.0)	1.5 (−2.3, 5.4)	0.21	↓	0.414
Oestrogens. oral	24	11.5 (8.0)	9.7 (6.2)	64	14.0 (14.0)	12.3 (12.4)	1.0 (−3.6, 5.5)	0.03	↓	0.612
Contraceptives (>50y)	24	5.1 (2.8)	5.9 (2.7)	64	3.2 (3.6)	6.5 (5.1)	2.5 (−0.9, 5.8)	0.31	↓	0.137
Hormone replacement therapy	24	38.7 (24.2)	36.3 (21.0)	64	31.1 (24.4)	29.8 (24.4)	2.6 (−9.7, 14.9)	0.60	↓	0.652
Clonidine	24	0.6 (1.5)	0.7 (2.7)	64	0.4 (1.5)	0.4 (1.6)	−0.3 (−1.4, 0.7)	0.11	↓	0.508 ^b^
Tibolon	24	3.6 (3.7)	3.8 (4.1)	64	2.6 (3.0)	2.3 (3.3)	−0.7 (−2.0, 0.6)	0.00	↓	0.294 ^b^

The desired direction of change is given in the column headed ‘Direction’. Numbers represent the number of DDDs prescribed per 6 months per 1000 patients

*n* = number of practices

^a^Adjusted difference between groups at end of intervention, corrected for baseline

^b^Statistically significant interaction

Thyroid dysfunctioning: Clonidine

P10: B (95% CI): 30.3 (15.1, 45.5). p: <0.001 P10: B (95% CI): 0.4 (−0.4, 1.2). p: 0.327

P90: B (95% CI): 186.7 (88.7, 284.7). p: <0.001 P90: B (95% CI):−1.3 (−2.2,−0.4). p: 0.005

Stomach complaints: Tibolon

P10: B (95% CI): 1.4 (−39.6, 42.6). p: 0.937 P10: B (95% CI): 0.9 (−0.8, 2.6). p: 0.297

P90: B (95% CI): 408.8 (341.1, 476.5). p: <0.001 P90: B (95% CI):−2.3 (−4.0,−0.6). p: 0.008

PPIs: Triple therapy (ATC A02BD04)

P10: B (95% CI): 91.5 (−120.0, 303.0). p: 0.378 P10: B (95% CI):−0.8 (−1.9, 0.3). p: 0.133

P90: B (95% CI): 347.4 (159.2, 0.8). p: <0.001 P90: B (95% CI): 1.4 (0.3, 2.5). p: 0.014

## Discussion

### Summary of the main findings

Our study found that the beneficial results obtained in earlier, well-controlled studies on audit and feedback with peer review in LQICs in primary care were not confirmed when we introduced this intervention in existing primary care LQICs. The per-protocol analyses showed that GPs from practices with the highest baseline volumes on test-ordering and prescribing showed the largest improvements.

Many participant and context related factors can be identified as possible explanations for the lack of overall effects of our intervention. Lack of confidence in and adherence to the strategy emerged during the trial; The origin and validity of the feedback was questioned by some participants, while others felt that this intervention was too complex and too ambitious. Although we provided complete transparency on the data sources and instructed the moderators in this respect, we learned from the process evaluation that the source of the feedback was often not clear to the participants [25]. We found that many groups failed to set achievable and measurable working agreements. More than half of the meeting reports we received from groups did not contain specific, achievable, realistic or measurable working agreements. What also seemed to have occurred is the phenomenon of choosing topics for quality improvement for which the group already showed good performance, the Sibley effect [33].

In a recently published article on how to provide feedback effectively Brehaut et al. provide 15 suggestions for designing and delivering effective feedback. [34] The feedback we provided meets all these suggestions but three. First providing the feedback as soon as possible at optimal intervals was not possible in our trial as we provided feedback on demand of the LQICs. Secondly we did not provide the feedback in more than one way, this could indeed have been helpful. The last suggestion we (partially) missed is to provide short key messages with an option to have extra detailed information on demand. This was impossible in this trial with the use of peer review as a means to discuss the feedback. Would we have provided key messages on individual feedback we would have impaired or at least influenced the peer review process, an essential part of LQIC work. When we look at the criteria for effective audit and feedback as defined by Ivers et al. in their somewhat older Cochrane review we conclude that we meet most criteria except including exclusively practices with poor baseline performance and the provision of predefined goals [35]. Including practices with poor baseline performance would have forced us to leave the stable and safe environment of the existing LQICs. The peer review effect where poorly performing GPs can learn from role models would have been impaired and the negative effects of an organisational reform would have occurred [8, 36, 37]. The provision of aggregated results from their own and neighbouring groups, together with the recommendations from clinical guidelines, can be regarded as implicit goal setting [21]. However we acknowledge that this differs to a greater extend from the definition of predefined goals suggested by Ivers et. al. [25].

We clearly underestimated the influence of a healthcare reform that was launched shortly after the start of this trial. As a result much specialist care was transferred to primary care, with Dutch GPs earning a higher income but at the same time feeling threatened in their autonomy and time management. Last but not least, the GPs are more than ever controlled by external parties such as the health inspectorate and healthcare insurers, which may have led to a more defensive attitude among GPs, resulting in higher test ordering rates.

### Strengths and limitations of the research methods used

Lack of power: our efforts to implement the strategy widely in the southern part of the Netherlands failed to recruit a sufficient number of groups for the trial, leaving us with an underpowered study. This could, in part, have been caused by the pragmatic character of our trial, with local pharmacists and experts on diagnostics leading the recruitment effort and moderating the groups. A major healthcare reform programme was launched shortly after our recruitment started [38], causing frustration among many GPs due to the resulting high administrative burden. This probably reduced their willingness to participate in our trial.

Choice of outcome and lack of quality indicators: we chose to express the volume of prescribed drugs in DDDs. A risk of this is that not all DDDs are compatible with the actual dosage physicians prescribe to a patient. For diclofenac, for instance, the normal dosage is 1.5 to 2 DDDs every day. However, this did not affect the comparability of the two groups, as both were affected by this form of distortion in the same way. If we had been able to provide feedback on quality indicators as well as volume data, a more valid insight into performance might have resulted, but with more interpretation problems for the GPs. Also, using volume data only has been proven to lower volumes especially in areas characterised by overuse [22].

Change of the protocol after its publication: the fact that all groups in the intervention arm, whether or not they had chosen a specific topic, were analysed using the intention-to-treat principle as if they had been exposed to the topic may have diluted the effects of the intervention. We would rather have analysed the effect of the intervention on changes in the direction of shared working agreements, as stated in the protocol, but as these were hardly established, we decided to use a per-protocol analysis as the second best option, after the protocol had been published.

Minimization of the Hawthorne effect: a strong point of the design we used is that it minimized the Hawthorne effect. On the downside, it might have caused contamination of the effects if we had not exposed all groups in the trial to the intervention at the same time [30, 39, 40].

#### Comparison with other studies

Evaluating the effect of large-scale implementation of a quality improvement strategy of proven effectiveness using a pragmatic design like ours has not often been performed. Earlier and ongoing work has focused mainly on one particular clinical topic (e.g., prescribing antibiotics for respiratory tract infections or X-rays for low back pain patients), while we applied the peer review strategy to a broad range of topics and focussed on both test ordering and prescribing behaviour [41–45]. By researching whether the results of more fully controlled trials were also found in large-scale implementation, we sought to contribute to the knowledge on ways to improve professional performance.

We are not aware of similar multi-faceted studies using audit and feedback with peer group discussion in this field that would allow direct comparison with our study, although much is known about the individual components we combined in our study.

Much work has been done on evaluating the effects of audit and feedback on both test ordering and prescribing behaviour in well-controlled trials. These interventions show a modest but statistically significant positive effect on changing professional behaviours but the heterogeneity of the trials prevents solid conclusions to be drawn [35, 41, 46–52]. Although audit and feedback on test ordering behaviour embedded in peer review in small groups has been found to be more effective than audit and feedback alone, it generally remains unclear exactly what factors contribute to this effect [10, 21, 53, 54]. The use of pragmatic designs in quality improvement research contributes to bridging the gap between academia and clinical practice [55, 56].

Multifaceted interventions like ours are complex by nature but seem attractive because the individual effects could add up. It remains unclear, however, whether multifaceted interventions or single interventions are more effective. Mostofian concluded in a review of reviews that multifaceted interventions are most effective in changing professional behaviour [57]. On the other hand, Irwin et al. concluded that there is no evidence for a larger effect of combined interventions, while Johnson and May find it likely that multifaceted interventions are more effective [52, 54].

Studies embedding the discussion of clinical topics in LQICs have reported a modest positive effect on prescribing costs and quality [14, 58–62]. Our finding, based on the per-protocol analysis, that groups with the highest volumes at baseline showed the largest improvement, is in line with the results presented by Irwin et al. [52].

#### Implications for future research

The problems on the fidelity of the feedback and with the uptake of the intervention could best be handled by assuring that a strong leader picks up the group and lead them forward. It may also be helpful to identify GPs with a low quality baseline performance representing an unwarranted deviation from the mean and target those GPs in this type of quality improvement initiatives. Other physicians who are already doing well can concentrate on what they are doing already; delivering high quality care. Further research is needed on whether low baseline performance is consistent behaviour for an individual GP. Also further research on the cut-off point for participants that can benefit from a quality improvement intervention like this is needed to clarify the population to be targeted best. Potential downsides of such an approach such as the loss of peer learning with learning from the best practices need to be addressed as well.

Further pragmatic research should be performed to confirm our findings that the results found in earlier well-controlled trials are not easily replicated. We therefore encourage other researchers to perform vigorous large scale evaluations of complex implementation strategies, preferably embedded and owned by the field, as we did

## Conclusions

Our intervention, which aimed at changing the test ordering and prescribing behaviour of GPs by means of auditing and feedback, embedded in LQICs, with academia at a distance, shows that the favourable results of earlier work could not be replicated. It appeared that large-scale uptake of evidence-based but complex implementation strategies with a minimum of influence of external researchers, but with the stakeholders in healthcare themselves being responsible for the work that comes with integrating this intervention into their own groups, was not feasible. Although our study suffered from a lack of power, we expect that even if a sufficient number of groups had been included, no clinically relevant changes would have been observed.

## Additional files

Additional file 1: Appendix 1. *Complete set of texts sent to GPs together with the feedback. Texts are pasted here in one document. (DOC 1843 kb)*

Additional file 2: Appendix 2. *Complete set of drugs and tests included in the databases for this intervention. (DOCX 33 kb)*

## Abbreviations

95% CI: 95% confidence interval
ATC: Anatomical Therapeutic Chemical Classification System
CME: Continuing medical education
DDD: Defined daily dosage
GP: General practitioner
LQIC: Local quality improvement collaborative
NHG: The Dutch College of General Practitioners
PTAM: Pharmacotherapeutic audit meeting
UTI: Urinary tract infection
