# Supplementary material for: Effect of audit and feedback with peer review on general practitioners’ prescribing and test ordering performance: a cluster-randomized controlled trial
Source: BMC Fam Pract. 2017 Apr 13;18:53. doi: 10.1186/s12875-017-0605-5 (PMC5390393; doi:10.1186/s12875-017-0605-5)
Supplement: Supplementary file 1 — Complete set of texts sent to GPs together with the feedback. Texts are pasted here in one document. (DOC 1843 kb) [file 12875_2017_605_MOESM1_ESM.doc]

**Appendix 1**

Complete set of texts sent to GPs together with the feedback. Texts are pasted here in one document.

Appendix for: Effect of audit and feedback with peer review in existing infrastructure in the south of the Netherlands on prescribing and test ordering performance of general practitioners after implementation: A cluster randomized controlled trial.[ISRCTN40008171]

J. Trietsch, B. v. Steenkiste, R. Grol, B. Winkens, H. Ulenkate, J. Metsemakers, T. van der Weijden

NHG = Dutch College of General Practitioners

**Module prostate complaints**

General
Difficulty in micturition is not an early symptom nor risk factor for prostate cancer.
Difficulty in micturition is caused by obstruction of the enlarged prostate, obstruction by smooth muscle of the bladder neck and/ or bladder dysfunction.
Prostatic hyperplasia can occur from the 3^rd^ decade. 90% of the patients with symptoms is 80 years and older. The size of the prostate does not correlate with the presence and severity of symptoms.
All of the following opinions and statements relate to men over 50 years of age.

Incidence and prevalence of BPH

|  | incidence (/1000 men) | Prevalence (/1000 men) |
| --- | --- | --- |
| General population of men | 2-4 | 4-6 |
| 45-64 years | 4-9 | 8-19 |
| 65-74 years | 8-24 | 24-33 |
| 75 years and older | 10-18 | 18-36 |

Because of the different definitions of BPH, there are large differences in incidence and prevalence between studies. In Dutch general practice the prevalence until 75 years is approx. 80/ 1000 patientyears, older than 75 years approx. 165/ 1000 patientyears.

**Part A, diagnostics**

General
In all patients with LUTS an underlying urinary tract infection should be excluded. Diagnosis and treatment of urinary tract infections are beyond the scope of this module.
There is no biochemical marker to diagnose BPH or prostate cancer nor to rule it out. The history and physical examination are the most important criteria to diagnose BPH.

**PSA** € 8,31

The prevalence of prostate cancer in the general population is 2-5%.
PSA is formed by the prostate epithelium and to a very small extent by other tissues. Increased by: prostate cancer, prostatitis, androgen use, BPH and perhaps after DRE and biopsies.
An abnormal DRE is an indication a referral to secondary care. Men whose prostate cancer is detected by screening have a slightly higher life expectancy than men with prostate cancer diagnosed otherwise.
An abnormal DRE and PSA <4 ng/ml results in 10-20% probability on prostate cancer.
A PSA 2-4 ng/ml gives a 15% chance of having prostate cancer. A PSA > 10ng/ml correlates with a probability of 10% to> 50% of prostate cancer.

**Free/Total PSA-ratio** € 8,31

This test increases the sensitivity of the PSA determination if its result is between 4-10mg / l. In this group the risk is as follows:

V/T PSA >0,25: 8% probability on prostate cancer being present

V/T PSA 0,20-0,25: 16% probability on prostate cancer being present

V/T PSA 0,15-0,20: 20% probability on prostate cancer being present

V/T PSA 0,10-0,15: 28% probability on prostate cancer being present

V/T PSA <0,10: 56% probability on prostate cancer being present

**Creatinine** € 1,41

In general malaise, with recurrent urinary tract infections or urinary creatinine levels should be determined. This allows the GFR to be calculated.
several methods are used to estimate the GFR. The Cockcroft-Gault and MDRD methods are the most used. Both are vulnerable to confounding factors (BMI, age, gender) and should be interpreted cautiously.
BPH can cause stowage and eventually renal failure.

*Source: NHG guideline M42, difficulties in micturition in the elderly men. NHG LESA rational test ordering 2006. Compass for diagnostics 2003*

**Module prostate complaints**

**Part B, pharmacotherapy**

general
Refer for invasive therapy if:

- The patient wishes it because of the severity of symptoms
- In recurrent acute urinary retentions
- In recurrent urinary tract infections
- When renal dysfunction is diagnosed and / or hydronephrosis
- When drug therapy has proven to be ineffective

Combination therapy of α1-receptor blockers with 5α-reductase inhibitors
has not been proven more effective than treatment with α1-receptor blockers alone.

**Alfuzosin** € 15,89 - € 26,92/month (1 DDD = 7,5 mg)

Drug of first choice according to the NHG guideline

**Tamsulosin** € 23,72/month (tablet), €12,36/month (caps) (1 DDD = 0,4 mg)

Drug of first choice according to the NHG guideline. Evidence is limited to trials with a short follow-up.

**Doxazosin** € 10,52 - € 13,15/month (1 DDD = 4 mg)

As effective as Alfuzosin and Tamsulosin, however non-compliance is higher due to side effects

**Terazosin** € 11,73 - € 12,11/month (1 DDD = 5 mg)

As effective as Alfuzosin and Tamsulosin, however non-compliance is higher due to side effects

**Prazosin** € 7,53/month (1 DDD = 5 mg)

Not mentioned in the NHG guideline

**Selective α1-receptor blockers**

Group of first choice according to the NHG guideline. Influences the muscle tone in the prostate and the urinary tract. The greatest effect is achieved within 2 weeks after the start of the therapy. The urinary flow improves by 20-30% this medication, the symptom score improves by 20%. These effects are independent of the size of the prostate. First administration may cause orthostatic hypotension. Severe hepatic impairment constitute a contraindication for the use of α1-receptor blockers

**5α-reductase blockers**

Slowing down the conversion of testosterone to dihydrotestosterone in the prostate. This results in less hyperplasia. In case of relatively large prostates (> 35-40ml), the risk for acute urinary retention and the need for a surgical intervention is reduced.
NHG guideline: not to be administered in primary care. Indicated when the prostate is large enough, in primary care the determination of the prostate size is not sufficiently reliable.

**Dutasteride** € 30,40/month (1 DDD = 0,5 mg)

The clinical effect is limited. Noticeable effect occurs after 12 months (subjective symptoms), effect on the flow can be detected earlier. The effect is noticeable with a prostate volume> 30ml but greater impact when > 40ml.

**Finasteride** € 26,09/month (1 DDD = 5 mg)

Improves symptom scores significantly. The clinical effect is limited in the short and medium term and fully reversible. In a small volume prostate symptom score improved no more than with placebo. Noticeable effect after 2-6 months.

*Source: NHG guideline M42, difficulties in micturition in the elderly men. Pharmacotherapeutic compass 2007.*

**Module chlamydia trachomatis**

general
PID: pelvic inflammatory disease, usually based on a salpingitis or adnexitis.
The incidence of Chlamydia trachomatis is 20 / 10,000.
The incidence of PID in women is 10 / 10,000
The incidence of PID in women caused by chlamydia is 5 / 10,000
In 13-40% PID leads to infertility (depending on number of infections) in 9% to EUG
The chance of infection after single or repeated contact is identical, 50-70%
The prevalence is 3-5% in young adults in the general population (<26years) and higher in young Surinamese, Antilleans and Arubans (up to 22%).
Incubation period: 1-3 weeks
After 1 year, 50% of the infections is no longer to traceable
After positive test: proactively perform partner detection up to 6 months prior to detection.

**Part A, diagnostics**

General

Physical examination with a suspected chlamydia often will yield no abnormalities. In the anamnesis also focus on anal and oropharyngeal complaints as well as complaints including post coital bleeding, spotting and abdominal complaints.

**DNA amplification, urine** € 44,59-88,79

Suitable for detecting Chlamydia in men. In women only suited for asymptomatic infections.

The test result remains positive until 3-4 weeks after completing a treatment successfully. (NHG guideline speaks of 2 weeks).

*Source: NHG guideline M82, the STD consultation. M50, PID. NHG LESA test ordering rationally 2006. CBO guideline STD and herpes neonatorum 2002. Diagnostic compass 2003. 2006 UK national guideline for the management of genital tract infection with chlamydia trachomatis. Sexually transmitted diseases treatment guidelines, 2006 CDC, Atlanta, VS.*

**DNA amplification, swab** € 44,59-88,79

Suited for collection in women. A swab of the cervix together with a swab from the urethra is preferred (2 swabs). This way an isolated urethritis is can’t be missed. An alternative for a cervical swab is a vaginal swab, this can be performed by the patient herself.

The test result remains positive until 3-4 weeks after completing a treatment successfully. (NHG guideline speaks of 2 weeks).

**IgG** € 8,31

Low sensitivity in asymptomatic infections. High sensitivity in active PID, pneumonia or lyfogranuloma venerum.

Test is specific to the genus, therefore the results can be difficult to interpret.

After the introduction of DNA amplification techniques, this test has become obsolete for detecting active chlamydia infection.

**Module chlamydia trachomatis**

**Part B, pharmacotherapy**

General

Treatment with a single dose: 7 days no sexual intercourse

Treatment with a 7 day regime: no sexual intercourse until the treatment has been finished

re-testing directly after the treatment has been finished has no value as the results remain positive for 2-4 weeks. Re-testing after 3-6 months is advisable. In 10% of the cases a re-infection occurs

**Azithromycin** € 9,05/ 1500mg (1DDD = 300 mg)

Drug of first choice according to the NHG guideline. A single administration of 1gr cures >95% of the infections. Is preferred over doxycycline because of a higher compliance rate. Can be administered during pregnancy (since august 2007). Therefore also drug of first choice in pregnant women.

**Doxycycline** €1,86-2,16/ week (1DDD = 100mg)

Drug of second choice according to NHG guideline. Treat for 7 days twice daily with 100mg. Do not prescribe to pregnant women.

**Amoxicillin** € 4,75/ 1125mg (1DDD = 1000 mg)

According to the NHG guideline, first choice in pregnant women. Treat 7 days 3 times daily with 500mg. 70% chance on cure of an infection.

NB: this advice is no longer valid, azithromycin has become drug of first choice in pregnancy as well.

**Erythromycin** €4,88-6,90/ 1500mg (1 DDD = 1000 mg)

Drug of second choice in pregnancy according to the NHG guideline , 4 times 500mg. also to be prescribed in cases of allergies. The chances of a successful treatment are 70%. In this case a re-test after 3 weeks is indicated.

*Source: NHG guideline M82, the STD consultation. M50, PID. CBO guideline STD and herpes neonatorum 2002. 2006 UK national guideline for the management of genital tract infection with chlamydia trachomatis. Sexually transmitted diseases treatment guidelines, 2006 CDC, Atlanta, VS. pharmacotherapeutic compass 2007.*

**Module rheumatic complaints**

notions

Prevalence RA open population: 10/ 1000

Prevalence RA GP, men: 2/ 1000

Prevalence RA GP, women: 5/ 1000

incidence RA: 1-2/ 1000/ year/ GP

Men: women: 1:3 (under 45 years) 5:6 (65+)

ACR criteria for the classification of RA (note: these are not diagnostic criteria!)

Main criteria (only valid when present more than 6 weeks)

1. Morning stiffness, longer than one hour
2. arthritis simultaneously present in three or more joint areas (left or right PIPs, MCPs, wrist, elbow, knee, ankle, MTPs)
3. arthritis in at least one hand joint: wrist, MCP or PIP
4. symmetric arthritis

secondary criteria

1. subcutaneously rheumatic nodules
2. serum rheumatoid factor (either test)
3. X-ray proven changes (X-hand/ wrist or foot)

A patient is said to have RA if 4 out of 7 criteria are satisfied

**part A, diagnostics**

general
Rheumatoid arthritis is a clinical diagnosis. For diagnosing rheumatoid arthritis diagnostic testing of little significance. For the differentiation of rheumatism and for instance gout testing might be important or when a patient does not fully meet the diagnostic criteria.

**serum rheumatoid factor**

Qualitative or semi-qualitative methods such as the latex fixation test and Waaler-Rose test are being replaced by the quantitative IgM test. The Waaler-Rose test adds nothing anymore.

Waaler-Rose € 11,19 (indication)

Latex fixation test € 7,62 (indicatief)

**IgM- rheuma factor** € 16,63

Quantitative test, test results of different laboratories are comparable. IgM-RF seems to fit better for criterion 6.

**Anti-CCP** price depends on laboratory

Antibody titer, the sensitivity matches IgM-RF sensitivity, has a very high specificity. In juvenile RA anti-CCP is often undetectable. NHG literature does not mention this test.

**ESR** € 1,41

ESR testing can sometimes help to differentiate between RA and a non-inflammatory disease. An elevated ESR makes it more likely that RA is present, a normal ESR does not exclude RA. An elevated ESR is especially useful in assessing disease activity.

**X-wrist/ foot/ hand** € 40,40 - 47,20

Indicated whit diagnostic uncertainty after taking the history and a careful examination and when serology tests return negative. The severity of radiological abnormalities correlate poorly with symptoms and functionality.

**CRP** € 3,88

Acute phase protein, can be elevated in chronical inflammatory diseases as well. ESR is a better proxy for the severity of the disease. CRP is also suitable for assessing disease activity. Testing ESR and CRP together adds nothing.

**AST** € 7,22

Measuring antistreptolysin levels is pointless when diagnosing RA.

Antibodies against group A, C of G streptococci are produced starting in the first week of the disease. Levels start to decrease after 6 weeks and return to normal levels within months. AST levels in healthy individuals are age dependent.

*Source: NHG guideline M41, rheumatoid arthritis. NHG LESA test ordering rationally 2006. Diagnostic compass 2003. National interdisciplinary guideline rheumatoid arthritis, NHG 2002.*

**Module rheumatic complaints**

**Part B, pharmacotherapy**

general
The GP will usually prescribe an NSAID when RA is suspected.
Initiating and monitoring DMARD prescriptions will usually be done by the rheumatologist. When a GP prolongs a prescription of DMARDs he or she shares the responsibility for the monitoring.

NSAIDs (cox-1) are mentioned as separate drugs on your own feedback. A part of this group is aggregated to a single group as the prescription volumes are very low.

Note: medication to protect the stomach is beyond the scope of this module.

**other NSAIDs (cox-1)** (DDDs depend on drug of choice)

None of the other NSAIDs is preferred. Based on past experience, for harm to the stomach and price the preference is for ibuprofen, diclofenac and naproxen.

**Indometacin/ Diclofenac** € 2,96-5,81/ 5,98-12,47/ mth (1DDD = 100mg/ 100mg)

Acetic acid derivates

Diclofenac is the least harmful for the stomach, except for ibuprofen, both are drugs of first choice. Indomethacin is comparable with naproxen considering the stomach

**Ibuprofen/ Naproxen** € 5,44-15,54/ 4,24-6,20/month (1DDD = 1200mg/ 500mg)

propionic acid derivates.

Ibuprofen is the least harmful to the stomach of all classic NSAIDs, with high dosages this advantage diminishes. Drug of first choice together with diclofenac.

Naproxen increases the risk for stomach bleeding more than ibuprofen and diclofenac.

**Meloxicam/ Nabumetone/ Piroxicam** € 9,32-14,26/ 10,03-15,56/ 5,77-9,86/ month (1DDD = 15mg/ 1000mg/ 20mg)

Are considered preferential cox-2 inhibitors because of their effects on COX-1 as well. In higher dosages COX-1 and COX-2 are inhibited. Ranking position of these drugs remains unclear.

Piroxicam is of the same group as meloxicam. EMEA limited the use and lowered the maximum dosage of piroxicam, never to be considered a drug of first choice.

**coxibs (cox-2)**

Celecoxib/ Etoricoxib/ Lumiracoxib/ Parecoxib € 25,40/ 31,63/ 23,51/238,50/ month (1DDD = 200mg/ 60mg/ 100mg/ 40mg)

Theoretically these drugs should cause less problems with the stomach and the kidneys. The evidence for this claim is limited.

Het European Medicines Agency (EMEA) considers the cardiovascular side effects of coxibs to be applicable to all coxibs . Coxibs are contraindicated in peripheral arterial vascular disease, ischemic heart diseases or with a history of CVA.

Parecoxib is only available for parenteral use. Experience with Lumiracoxib is limited. It seems to be suited for administration in arthrosis.

**Diclofenac with Misoprostol** € 17,78-23,22 (combination: no DDD given)

misoprostol 800μg/day has proven to be more effective than protonpump inhibitors or H_2_-antagonists in a doubled dosage. 800μg/day leads to much gastrointestinal side effects.

For the prevention of stomach ulcers in NSAID usage this combination is a sound option. The recommended dosage is: 3 times daily 1 tablet diclofenac/ misoprostol (50mg/200μg)

**DMARD’s** (DDDs depend on drug of choice)

Most DMARDs are to be administered only by a specialist with specific knowledge and experience on these drugs. GPs however can be confronted with questions on side effects, interactions or the request for prolongation of these drugs. GPs should at least know and recognize the side effects of DMARDs.

Rheumatologists are to be consulted easily with questions concerning usage of DMARDs

| **common DMARDs:** | **Side effects** | **Interactions/ warnings** |
| --- | --- | --- |
| Sulfasalazine | Gastrointestinal complaints, headache, rash, disturbance of liver function, depressive feelings, leukocytopenia, agranulocytosis |  |
| Methotrexate | Gastrointestinal complaints, disturbance of liver function, leukocytopenia, thrombocytopenia, pneumonitis, , stomatitis, subcutaneous nodules | Trimethoprim containing drugs strongly increase the risk for leukocytopenia.  Up to 3 month after ceasing use: ♀avoid getting pregnant, ♂ avoid causing pregnancy |
| Leflunomide | Gastrointestinal complaints, disturbance of liver function, leukocytopenia, hypertension | Up to 2 years after ceasing use: ♀ avoid getting pregnant, ♂ avoid causing pregnancy |
| Etanercept, Infliximab  Adalimumab | No specific side effects. Possible increased risk for malignancies, remains unclear. | Increased susceptibility for TBC, risk for reactivation of latent TBC. Mild bacterial infections can suddenly turn into severe disease.. **always consult a rheumatologist in these cases.** |
| Gold preparations, d-Penicillamine | Gastrointestinal complaints, leukocytopenia, thrombocytopenia, proteinuria, stomatitis, dermatitis |  |
| (Hydroxy)chloroquine | Retinal disorders in long lasting higher dosages, intolerance for sunlight |  |
| Azathioprine | Gastrointestinal complaints, leukocytopenia, thrombocytopenia, influenza like disease, disturbance of liver function | Up to 3 month after ceasing use: ♀avoid getting pregnant, ♂ avoid causing pregnancy |
| Ciclosporin | Disturbances of kidney function, hypertension, hypertrichosis, gingival hyperthrophy |  |

*Source: NHG guideline M41, reumatoid artritis. Pharmacotherapeutic compass 2007. Pharmacotherapeutic directive pain relief, NHG 2005. updated consensus statement on biological agents for the treatment of rheumatic diseases, 2007. DE Furst, FC Breedveld et.al. Ann Rheum Dis 2007;66;2-22.*

**Module menopausal symptoms**

notions
Perimenopause: period from the start of irregular menses until 1 year after the last

menses.
Post-menopause: means the period commencing one year after the last menses.

At the age of 51 years, has an average of 50% of the women reached menopause. Smoking women enter menopause an average of 1-1½ years earlier, women without a uterus average 1-2 years earlier.

Incidence perimenopausal symptoms: 10/1000 women / year
Prevalence perimenopausal symptoms: 80/1000 women / year (category: 54-64 yrs)
Average duration of symptoms: 4 years (range: a few months - 11 years)

**Part A, diagnostics**

General advice
Diagnose menopause is set on the basis of the anamnesis, **laboratory diagnostics adds nothing.**
In the diagnosis of menopausal symptoms, it is important to rule out other causes before diagnosing transition. Thyroid dysfunction and pregnancy can provide the same pattern of symptoms as the transition.

**LH** € 6,65

Fluctuates during the cycle. Longitudinal studies showed the LH not to be a good parameter for the transition biochemically.

Even in women with no uterus LH testing is not indicated.

**Estradiol** € 8,31

Fluctuates during the cycle. Longitudinal studies showed estradiol not to be a good parameter for the transition biochemically. Even in women with no uterus testing Estradiol levels is not indicated.
When premature ovarian failure (<40 years) is suspected testing estradiol levels could be useful.

**FSH** € 6,65

Fluctuates during the cycle, rising gradually during life, in the menopause a stronger increase. Unsuitable in order to determine or to exclude the biochemical menopause at the individual level. After determination of a high FSH level normal menstruation may still occur. Even in women with no uterus FSH testing is not indicated.
Testing FSH levels is indicated for the diagnosis of primary or secondary amenorrhea.

*Source: NHG guideline M73, perimenopause. NHG LESA test ordering rationally 2006. Compass for diagnostics 2003.*

**Module menopausal symptoms**

**Part B, pharmacotherapy**

general
The transition-related complaints of vaginal dryness and decrease libido seem related to lower estrogen levels. Explanation of this relation and the advice to take more time for foreplay and possibly the use of lubrificantia may be a sensible advice to use.
Local estrogen (eg Synapause) are effective without an increased risk of cardiovascular disease, carcinoma of the breasts or endometrium. The risk of urinary tract infections also decreases with its use.

**Estrogen alone** (various formulations)
Highly effective against vasomotor symptoms resulting of the transition. Usage lowers the risk of fractures.
A significant increase in the risk of CVD and thrombosis is present when started after the menopause (especially in the first year of use).
Unopposed estrogen therapy increases the risk of endometrial carcinoma and possibly reduces the risk of breast cancer.
Recently evidence was published showing that these agents also increase the risk of ovarian cancer.

**Combination preparations** (various formulations)
Highly effective against vasomotor symptoms occurring as a result of the transition. The risk of fractures and colorectal cancer is reduced when using these drugs. 3 years after discontinuation of the medication, this effect will have disappeared. The risk of breast cancer is increased until 3 years after stopping these drugs, however the mortality appears not to be increased.
The risk of endometrial cancer is not changed (in sequential administration) or is decreased (in continuous administration). The risk of cardiovascular disease and thrombosis is increased until 3 years after discontinuation.

**Tibolone** € 18,74/ month (1 DDD = 2,5 mg)

Steroid with estrogenic, progestagenic and androgenic activity. Effective against vasomotor symptoms as a result of the transition. Works optimally after a few months. Is indicated for symptoms in the postmenopausal period. Use of tibolone increases the risk of occurrence of endometrial carcinoma.
The risk for breast cancer risk has not been established in well-designed research. It may improve sexual function something.

**contraceptives** (oral and others) in women> 50 years
Not indicated as a therapy against symptoms consistent with the transition not even when continued from the pre-menopause. When a woman is using contraceptives already it seems sensible to agree on an age when usage will be stopped beforehand.

**Clonidine** € 7,67/ month (1 DDD = 0,45 mg)

Centrally acting antihypertensive agent. Only limited research is available. Clonidine might be effective against vasomotor symptoms as a result of the transition. Caution should be taken when given in combination with other antihypertensive drugs.

ß-blockers can lead to a rebound hypertension after discontinuation of clonidine when prescribed at the same time. Usual dosage: 3 times 0,050mg daily.

Management of menopausal symptoms, benefits and risks.

|  | Effect on vasomotor symptoms | Risk of fractures | Risk of CVD | Risk of breast cancer | Risk of endometrial cancer | Risk of ovarian cancer | Risk of colon carcinoma | Risk of thrombosis |
| --- | --- | --- | --- | --- | --- | --- | --- | --- |
| Estrogen alone | ++ | ↓ | ↑^1^ | ↓? | ↑^2^ | ↑? |  | ↑ |
| Combination preparations | ++ | ↓^8^ | =^5^ | ↑ | =3/ ↓^4^ |  | ↓^7^ | =^6^ |
| Tibolone | + |  |  | ↑? | ↑ |  |  |  |
| Clonidine | + |  |  |  |  |  |  |  |

↓: decrease ^1^ when started after menopause, especially during the first year

↑: increase ^2^ with the uterus in situ

=: unchanged ^3^ sequential administration

?: effects unclear ^4^ continuous administration

++: large improvement ^5^ 3 years after discontinuation, first 3 years increased risk

+: small improvement ^6^ 3 years after discontinuation, first 3 years increased risk

^7^ 3 years after discontinuation a return to placebo risk levels

^8^ risk seems to return to baseline levels after 3 years

Clear boxes: no statistically significant effects found or has not been researched yet.

*Source: NHG guideline M73, perimenopause. NHG manifest on contraceptive use in the menopause, june 2006. Pharmacotherapeutic compass 200*7. *Gerardo Heiss et al.* ***Health Risks and Benefits 3 Years After Stopping Randomized Treatment With Estrogen and Progestin,*** JAMA*2008;299(9):1036-1045*

**Module dyslipidemia**

Notions:
Estimation of cardiovascular risk in the absence of CVD, and in the absence of DM2 is done using the SCORE table

Estimation of the cardiovascular risk in the presence of DM2 is done on the basis of the UKPDS risk engine; free download from: http://www.dtu.ox.ac.uk/

Assessing the mortality risk after a previous cardiovascular event is not covered in the guideline cardiovascular risk.

**Part A, diagnostics**

General

Laboratory tests for cholesterol and triglycerides are prone to errors. The most reliable results are obtained after 12 hours of fasting and in repeated measurements after 1-8 weeks. For determination of the risk profile of a patient a single measurement may be sufficient.

**Total cholesterol** € 1,41

Important for calculating the risk. Total cholesterol is reduced 10-20% after surgery, trauma or other acute stress (such as myocardial infarction).

**Homocysteine** € 38,80

Hyperhomocysteinemia increases the risk of CVD. The elevated risk is similar to that of high cholesterol levels and smoking. Recent studies however show no effect on cardiovascular events after treatment of hyperhomocysteinemia. The diagnostic value therefore is still limited to the refinement of the risk profile.

**HDL** € 2,77

Important for calculating the risk. A relative limit applied by the NHG is 0,8mmol / l.
Reference values of women are 0.2-0.3 mmol / l higher than that of men.

**Triglycerides** € 1,89

Test results are highly dependent on factors such as exercise, type of food, time since last meal and prescribed medication (estrogens, corticosteroids, thiazides, propranolol, chlorthalidone). It is needed for calculating LDL levels when direct assay of LDL levels is not possible. Not a necessary test for the risk assessment.

**LDL** € 6,07

Usually indirect assay, is calculated by the laboratory on the basis of the Friedewald formula. If the triglyceride level is higher than 4.5, this formula is not reliable. The direct assessment is relatively insensitive to interference by high triglyceride levels.
This value is used to monitor the treatment, preferably a few weeks after the start of the therapy or no later than after 3 months.

**Total cholesterol/ HDL** r**atio**

This test is needed for assessing the mortality risk in accordance with the score table (see below).

| Score table for calculating the mortality risk for patients without prior CVD and DM2 | | | | | | | | | | | | | | | | | | | | | | | | | | | | | |
| --- | --- | --- | --- | --- | --- | --- | --- | --- | --- | --- | --- | --- | --- | --- | --- | --- | --- | --- | --- | --- | --- | --- | --- | --- | --- | --- | --- | --- | --- |
|  | |  | | |  | | | | | | |  | | | | | | | | | | |  | | |  |  |  |  |
|  | |  |  | | **Women** | | | | | | |  | | | |  | | |  | | | | **Men** | | |  |  |  |  |
|  | |  |  | | ***Non smoking*** | | | |  | |  | ***smoking*** | | | |  | | | ***Non smoking*** | | | |  |  |  |  | ***smoking*** |  |  |
| **SBP** | |  | | |  | | | | | | | **Age** | | | | | | | | | | |  | | |  |  |  |  |
| 180 | |  | 8 | | 10 | 11 | 13 | 14 |  | | 15 | 18 | 20 | 23 | 26 |  | | | 13 | 15 | 17 | 20 | 22 |  | 23 | 27 | 31 | 35 | 38 |
| 160 | |  | 8 | | 7 | 8 | 9 | 10 |  | | 11 | 13 | 15 | 17 | 19 | 65 | | | 9 | 11 | 13 | 14 | 16 |  | 17 | 20 | 23 | 26 | 29 |
| 140 | |  | 4 | | 5 | 8 | 7 | 7 |  | | 8 | 9 | 11 | 12 | 14 |  | | | 6 | 8 | 9 | 10 | 12 |  | 12 | 15 | 17 | 19 | 21 |
| 120 | |  | 3 | | 3 | 4 | 5 | 5 |  | | 5 | 7 | 8 | 9 | 10 |  | | | 5 | 6 | 7 | 7 | 8 |  | 9 | 11 | 12 | 14 | 16 |
| 180 | |  | 4 | | 5 | 6 | 7 | 8 |  | | 8 | 10 | 11 | 13 | 14 |  | | | 7 | 9 | 10 | 12 | 13 |  | 14 | 16 | 19 | 21 | 24 |
| 160 | |  | 3 | | 4 | 4 | 5 | 5 |  | | 6 | 7 | 8 | 9 | 10 | 60 | | | 5 | 6 | 7 | 8 | 9 |  | 10 | 12 | 14 | 16 | 17 |
| 140 | |  | 2 | | 3 | 3 | 3 | 4 |  | | 4 | 5 | 6 | 7 | 7 |  | | | 4 | 5 | 5 | 6 | 7 |  | 7 | 9 | 10 | 11 | 13 |
| 120 | |  | 1 | | 2 | 2 | 2 | 3 |  | | 3 | 3 | 4 | 5 | 5 |  | | | 3 | 3 | 4 | 4 | 5 |  | 5 | 6 | 7 | 8 | 9 |
| 180 | |  | 2 | | 3 | 3 | 4 | 4 |  | | 4 | 5 | 6 | 7 | 8 |  | | | 4 | 5 | 6 | 7 | 8 |  | 8 | 10 | 11 | 13 | 15 |
| 160 | |  | 2 | | 2 | 2 | 3 | 3 |  | | 3 | 4 | 4 | 5 | 5 | 55 | | | 3 | 4 | 4 | 5 | 6 |  | 6 | 7 | 8 | 9 | 11 |
| 140 | |  | 1 | | 1 | 2 | 2 | 2 |  | | 2 | 3 | 3 | 3 | 4 |  | | | 2 | 3 | 3 | 4 | 4 |  | 4 | 5 | 6 | 7 | 8 |
| 120 | |  | 1 | | 1 | 1 | 1 | 1 |  | | 1 | 2 | 2 | 2 | 3 |  | | | 2 | 2 | 2 | 3 | 3 |  | 3 | 4 | 4 | 5 | 5 |
| 180 | |  | 1 | | 1 | 2 | 2 | 2 |  | | 2 | 3 | 3 | 4 | 4 |  | | | 3 | 3 | 4 | 4 | 5 |  | 5 | 6 | 7 | 8 | 9 |
| 160 | |  | 1 | | 1 | 1 | 1 | 2 |  | | 2 | 2 | 2 | 3 | 3 | 50 | | | 2 | 2 | 3 | 3 | 3 |  | 4 | 4 | 5 | 6 | 6 |
| 140 | |  | 1 | | 1 | 1 | 1 | 1 |  | | 1 | 1 | 2 | 2 | 2 |  | | | 1 | 2 | 2 | 2 | 2 |  | 3 | 3 | 4 | 4 | 5 |
| 120 | |  | 0 | | 1 | 1 | 1 | 1 |  | | 1 | 1 | 1 | 1 | 2 |  | | | 1 | 1 | 1 | 2 | 2 |  | 2 | 2 | 3 | 3 | 3 |
| 180 | |  | 1 | | 1 | 1 | 1 | 1 |  | | 1 | 1 | 2 | 2 | 2 |  | | | 1 | 1 | 1 | 1 | 2 |  | 2 | 2 | 2 | 3 | 3 |
| 160 | |  | 0 | | 0 | 1 | 1 | 1 |  | | 1 | 1 | 1 | 1 | 1 | 40 | | | 1 | 1 | 1 | 1 | 1 |  | 1 | 2 | 2 | 2 | 2 |
| 140 | |  | 0 | | 0 | 0 | 0 | 1 |  | | 1 | 1 | 1 | 1 | 1 |  | | | 0 | 1 | 1 | 1 | 1 |  | 1 | 1 | 1 | 1 | 2 |
| 120 | |  | 0 | | 0 | 0 | 0 | 0 |  | | 0 | 0 | 1 | 1 | 1 |  | | | 0 | 0 | 0 | 1 | 1 |  | 1 | 1 | 1 | 1 | 1 |
|  | | 4 5 6 7 8 4 | | | | | | | | | | 5 6 7 8 4 5 6 7 8 4 5 6 7 8 | | | | | | | | | | | | | | | | | |
|  | |  | | | | | | | | | | **Total cholesterol/HDL-cholesterol ratio** | | | | | | | | | | | | | | | | | |
|  |  |  | | 0 – 4% mortality risk by CVD | | | | | |  | | 5 – 9% mortality risk by CVD | | | | |  | ≥10% mortality risk by CVD | | | | | | | | | | | |
|  |  |  | |  | | | | | |  | |  | | | | |  |  | | | | | | | | | | | |

*Source: NHG guideline M84, cardiovascular risk management version 1.3 (2007). NHG LESA test ordering rationally 2006. Diagnostic compass 2003.*

**Module Dyslipidemia**

**Part B, pharmacotherapy**

General

Initiate treatment when:

- In patients without a history of CVD or DM2 with a 10-year mortality risk over 10% in combination with LDL level > 2,5 mmol/l. When additional risk factors are present a lower mortality risk can justify initiation of treatment.

- In patients with a history CVD and/ or DM2 with LDL level > 2,5 or with a history of DM2 and a total cholesterol level > 4,5 mmol/l. When additional risk factors are present a lower mortality risk can justify initiation of treatment.

additional risk factors : - family history of CVD

- unhealthy diet

- little exercise

- BMI > 30 kg/ m^2^

- waist circumference > 102 cm (♂) or 88 cm (♀)

In young patients with a history of DM2 and a favorable risk profile, higher treatment thresholds are held.

Therapy objectives:

- in patients without CVD or DM2: LDL < 2,5 mmol/l or a decrease of 1,0 mmol/l.

- In patients with CVD or DM2: LDL <2,5 mmol/l.

**Pravastatin** € 14,52 - 19,68 (selektine) / month based on 20mg (20mg = 1DDD)

Drug of first choice according to the NHG guideline (along with others). Proven to be effective on hard endpoints. Initial dose 1dd 40mg.

**Atorvastatin** € 22,92/ month based on 10mg (10mg = 1DDD)

Mentioned in the NHG guideline. As effective as simvastatin and pravastatin.

Without a history of CVD and/ or DM2 not advised (NHG/ CBO).

**Simvastatin** € 8,31/ 11,13 (zocor) / month based on 15mg (15mg = 1DDD)

Drug of first choice according to the NHG guideline (along with others). Proven to be effective on hard endpoints. Initial dose 1dd 40mg.

**Fluvastatin** € 17,14/ month based on 40mg (40mg = 1DDD)

Mentioned in the NHG guideline, less effective in preventing new cardiovascular events.

Without a history of CVD and/ or DM2 not advised (NHG/ CBO).

**Rosuvastatin** € 23,93/ month based on 10mg (10mg = 1DDD)

Not yet proven to be effective on hard clinical endpoints. Only to be used when target cholesterol levels are not reached with other drugs .

Without a history of CVD and/ or DM2 not advised.

**Bezafibrate** € 10,92/ month based on 600mg (600mg = 1DDD)

Increases HDL levels, not yet proven to be effective on hard clinical endpoints.

**Ciprofibrate** € 19,66 - 38,06 (Hyperlipen) / month based on 100mg (100mg = 1DDD)

Increases HDL levels, not yet proven to be effective on hard clinical endpoints.

**Gemfibrozil** € 13,93/ month based on 1200mg (1200mg = 1DDD)

Increases HDL levels, not yet proven to be effective on hard clinical endpoints.

**Acipimox** € 26,90/ month based on 500mg (500mg = 1DDD)

Nicotic acid analog. Increases HDL levels, not yet proven to be effective on hard clinical endpoints.

**Ezetimibe** € 37,45/ month based on 10mg (10mg = 1DDD)

Increases HDL levels, not yet proven to be effective on hard clinical endpoints.

Decreases the reception of cholesterol and plant sterols from the small intestine.

**Nicotic acid** € 39,97/ month based on 2000mg (2000mg = 1DDD)

vitamin B7. Increases HDL levels, not yet proven to be effective on hard clinical endpoints.

**Xantinol nicotinate** € 8,76/ month based on 900mg (900mg = 1DDD)

Increases HDL levels, not yet proven to be effective on hard clinical endpoints. Is dissuaded in the pharmacotherapeutic compass for pharmacotherapeutic reasons because of insufficient proof of effectiveness.

**Colestyramine** € 33,51/ month based on 14g (14g = 1DDD)

Decreases the reception of cholesterol through the intestine. Increases HDL levels, not yet proven to be effective on hard clinical endpoints.

**Ezetimibe/ Simvastatin** € 46,85 – 57,78/ month

Nicotic acid analog combined with a statin. No convincing proof for effectiveness on hard clinical endpoints considering ezetimibe. The pharmacotherapeutic compass has not yet evaluated the effectiveness of this combination. 1 DDD = 1 tablet regardless of dosage.

| Score table for calculating the mortality risk for patients without prior CVD and DM2 | | | | | | | | | | | | | | | | | | | | | | | | | | | | | |
| --- | --- | --- | --- | --- | --- | --- | --- | --- | --- | --- | --- | --- | --- | --- | --- | --- | --- | --- | --- | --- | --- | --- | --- | --- | --- | --- | --- | --- | --- |
|  | |  | | |  | | | | | | |  | | | | | | | | | | |  | | |  |  |  |  |
|  | |  |  | | **Women** | | | | | | |  | | | |  | | |  | | | | **Men** | | |  |  |  |  |
|  | |  |  | | ***Non smoking*** | | | |  | |  | ***smoking*** | | | |  | | | ***Non smoking*** | | | |  |  |  |  | ***smoking*** |  |  |
| **SBP** | |  | | |  | | | | | | | **Age** | | | | | | | | | | |  | | |  |  |  |  |
| 180 | |  | 8 | | 10 | 11 | 13 | 14 |  | | 15 | 18 | 20 | 23 | 26 |  | | | 13 | 15 | 17 | 20 | 22 |  | 23 | 27 | 31 | 35 | 38 |
| 160 | |  | 8 | | 7 | 8 | 9 | 10 |  | | 11 | 13 | 15 | 17 | 19 | 65 | | | 9 | 11 | 13 | 14 | 16 |  | 17 | 20 | 23 | 26 | 29 |
| 140 | |  | 4 | | 5 | 8 | 7 | 7 |  | | 8 | 9 | 11 | 12 | 14 |  | | | 6 | 8 | 9 | 10 | 12 |  | 12 | 15 | 17 | 19 | 21 |
| 120 | |  | 3 | | 3 | 4 | 5 | 5 |  | | 5 | 7 | 8 | 9 | 10 |  | | | 5 | 6 | 7 | 7 | 8 |  | 9 | 11 | 12 | 14 | 16 |
| 180 | |  | 4 | | 5 | 6 | 7 | 8 |  | | 8 | 10 | 11 | 13 | 14 |  | | | 7 | 9 | 10 | 12 | 13 |  | 14 | 16 | 19 | 21 | 24 |
| 160 | |  | 3 | | 4 | 4 | 5 | 5 |  | | 6 | 7 | 8 | 9 | 10 | 60 | | | 5 | 6 | 7 | 8 | 9 |  | 10 | 12 | 14 | 16 | 17 |
| 140 | |  | 2 | | 3 | 3 | 3 | 4 |  | | 4 | 5 | 6 | 7 | 7 |  | | | 4 | 5 | 5 | 6 | 7 |  | 7 | 9 | 10 | 11 | 13 |
| 120 | |  | 1 | | 2 | 2 | 2 | 3 |  | | 3 | 3 | 4 | 5 | 5 |  | | | 3 | 3 | 4 | 4 | 5 |  | 5 | 6 | 7 | 8 | 9 |
| 180 | |  | 2 | | 3 | 3 | 4 | 4 |  | | 4 | 5 | 6 | 7 | 8 |  | | | 4 | 5 | 6 | 7 | 8 |  | 8 | 10 | 11 | 13 | 15 |
| 160 | |  | 2 | | 2 | 2 | 3 | 3 |  | | 3 | 4 | 4 | 5 | 5 | 55 | | | 3 | 4 | 4 | 5 | 6 |  | 6 | 7 | 8 | 9 | 11 |
| 140 | |  | 1 | | 1 | 2 | 2 | 2 |  | | 2 | 3 | 3 | 3 | 4 |  | | | 2 | 3 | 3 | 4 | 4 |  | 4 | 5 | 6 | 7 | 8 |
| 120 | |  | 1 | | 1 | 1 | 1 | 1 |  | | 1 | 2 | 2 | 2 | 3 |  | | | 2 | 2 | 2 | 3 | 3 |  | 3 | 4 | 4 | 5 | 5 |
| 180 | |  | 1 | | 1 | 2 | 2 | 2 |  | | 2 | 3 | 3 | 4 | 4 |  | | | 3 | 3 | 4 | 4 | 5 |  | 5 | 6 | 7 | 8 | 9 |
| 160 | |  | 1 | | 1 | 1 | 1 | 2 |  | | 2 | 2 | 2 | 3 | 3 | 50 | | | 2 | 2 | 3 | 3 | 3 |  | 4 | 4 | 5 | 6 | 6 |
| 140 | |  | 1 | | 1 | 1 | 1 | 1 |  | | 1 | 1 | 2 | 2 | 2 |  | | | 1 | 2 | 2 | 2 | 2 |  | 3 | 3 | 4 | 4 | 5 |
| 120 | |  | 0 | | 1 | 1 | 1 | 1 |  | | 1 | 1 | 1 | 1 | 2 |  | | | 1 | 1 | 1 | 2 | 2 |  | 2 | 2 | 3 | 3 | 3 |
| 180 | |  | 1 | | 1 | 1 | 1 | 1 |  | | 1 | 1 | 2 | 2 | 2 |  | | | 1 | 1 | 1 | 1 | 2 |  | 2 | 2 | 2 | 3 | 3 |
| 160 | |  | 0 | | 0 | 1 | 1 | 1 |  | | 1 | 1 | 1 | 1 | 1 | 40 | | | 1 | 1 | 1 | 1 | 1 |  | 1 | 2 | 2 | 2 | 2 |
| 140 | |  | 0 | | 0 | 0 | 0 | 1 |  | | 1 | 1 | 1 | 1 | 1 |  | | | 0 | 1 | 1 | 1 | 1 |  | 1 | 1 | 1 | 1 | 2 |
| 120 | |  | 0 | | 0 | 0 | 0 | 0 |  | | 0 | 0 | 1 | 1 | 1 |  | | | 0 | 0 | 0 | 1 | 1 |  | 1 | 1 | 1 | 1 | 1 |
|  | | 4 5 6 7 8 4 | | | | | | | | | | 5 6 7 8 4 5 6 7 8 4 5 6 7 8 | | | | | | | | | | | | | | | | | |
|  | |  | | | | | | | | | | **Total cholesterol/HDL-cholesterol ratio** | | | | | | | | | | | | | | | | | |
|  |  |  | | 0 – 4% mortality risk by CVD | | | | | |  | | 5 – 9% mortality risk by CVD | | | | |  | ≥10% mortality risk by CVD | | | | | | | | | | | |
|  |  |  | |  | | | | | |  | |  | | | | |  |  | | | | | | | | | | | |

*Source: NHG guideline M84, cardiovascular risk management version 1.3 (2007). pharmacotherapeutic compass 2007.*

**Module Diabetes Mellitus type 2**

notions
This module does not have the intention to cover the entire field of cardiovascular risk management. Please refer to the standard cardiovascular risk of the NHG for more information.

Reference values for the diagnosis of diabetes mellitus and impaired fasting glucose (mmol/l). capillary blood venous plasma

normal glucose fasting <5,6 <6,1

glucose random <7,8 <7,8

impaired glucose fasting >5,6 en <6,0 >6,1 en <6,9

diabetes mellitus glucose fasting >6,0 >6,9

glucose random >11,0 >11,0

prevalence: 36/1000 men, 39/1000 women (type 1 en 2)

2-4 times higher in: Moroccan, Turkish and Surinamese descent. Hindustani descent: prevalence even higher and more micro and macro vascular complications.

Incidence: 4,5/ 1000/ year, increasing with age.

**Part A, diagnostics**

general
Well calibrated portable meters can have a deviation of 10-15%
Different methods are used to estimate kidney function. The Cockcroft-Gault and MDRD are used most frequently. Both are prone to confounders (eg. BMI, age, sex) and need cautious interpretation.

Note: all prices mentioned are indicative only for laboratory testing

**Glucose random** € 1,41

To be determined with complaints. Furthermore determine every three years in each clinic visitor> 45 years: - when DM2 is present with parents/brother/ sister

- with a history of hypertension

- with a history of CVD

- with a history of dyslipaedemia

- from Turkish, Moroccan of Surinamese descent

- from Hindustani descent ( >35 yr),

- when BMI >27,

- with a history of diabetes gravidarum.

Laboratory results for random glucose are lower with a higher hematocrit present.

May alternatively be assayed instead of fasting glucose when following-up a patient. These values are not interchangeable.

**Fasting glucose** € 1,41

Mandatory for diagnosing when random glucose <11,0. First step after diagnosing DM2 is lifestyle advice. Oral medication is only to be adjusted after testing fasting glucose levels. To be tested once every three months after diagnosing DM2 and initiating drug treatment.

Laboratory results for fasting glucose are lower with a higher hematocrit present.

**HbA1c** € 6,65

To be tested after diagnosing DM2. Plays no role according to international agreements for diagnosing DM2. To be tested once a year to follow-up on the treatment. Target HbA1c levels: <7%.

Only valid parameter to monitor whether the initiated therapy is effective. False increased levels can be seen with erythrocyte abnormalities, elevated urea or overuse of aspirin.

**Total cholesterol** € 1,41

Determine after diagnosing DM 2 and annually thereafter. Target level is <4.5 mmol / l in DM 2 if the LDL cannot be determined because of high triglyceride levels.

**HDL** € 2,77

Determine after diagnosing DM 2 and annually thereafter. Target level is > 1,0 mmol/L

**creatinine** € 1,41

Determine after diagnosing DM 2. This is used to calculate the kidney function, see note above (under "general").

**Albumin concentration, urine** € 1,41

To be tested annually. Abnormal values should be confirmed by a second assay a few months later. The target value is <20mg / ml. There is a slight preference determining the albumin / creatinine ratio in the urine.

*Source: NHG guideline M01, diabetes mellitus type 2. NHG LESA test ordering rationally 2006. Diagnostic compass 2003.*

**LDL** € 6,07

LDL is in many cases no laboratory determination in the strict sense, it is calculated on the basis of total cholesterol, triglycerides and HDL.
Determine after diagnosing DM 2 and annually thereafter. A HbA1c> 8.5 leads to underestimation of LDL levels. Policy should then be determined on the basis of total cholesterol. In the case of a high triglyceride level, it is impossible to determine LDL levels. Target level is <2.5 mmol / l in diabetics.

**Triglycerides** € 1,89

Determine after diagnosing DM 2 and annually thereafter. Variation on the same person can be up to 30%, both in the long term and the short term (day). Increases were observed up to 12 hours after ingestion of food.

**Module Diabetes Mellitus type 2**

**Part B, pharmacotherapy**

General

NHG advices to start treatment with three months lifestyle management. If this effect is insufficient treatment with medication should be initiated. The policy should be adjusted based upon the fasting blood glucose and HbA1c. Determination of HbA1c is most useful to determine whether the proposed targets are achieved or to check if a new step is indicated in the policy.

**Glibenclamide** € 4,70-8,16/ month (10mg = 1DDD)

Relatively high risk of hypoglycemia, administration is not recommended. See further text under sulfonylureas.

**sulfonylureas** (excl. glibenclamide)

Promoting insulin release from β-cells. Reducing incidence of microvascular complications has not been proven, mortality is not reduced. Use of sulfonylureas can result in a weight gain of 2-5 kg. There is no significant difference demonstrated between the different sulfonylureas with the exception of glibenclamide.

Glicazide € 6,15-7,74/ month (0,16gr = 1DDD)

Glimepiride € 4,83-5,11/ month (2mg = 1DDD)

Tolbutamide € 2,44/ month (1,5gr = 1DDD)

**Metformin** € 3,81-4,76/ month (2gr = 1DDD)

Drug of first choice. Inhibits glucose production in the liver and enhances peripheral insulin sensitivity. In high doses metformin has favorable effects on total cholesterol and LDL.

**Thiazolidinediones**

Improving peripheral insulin sensitivity. In overweighed patients both drugs lower glucose levels and insulin, triglycerides and fatty acids. Liver function disorders are a relative contraindication as is heart failure. Based on experience cost and proven effectiveness of metformin this remains the drug of first choice.

**Pioglitazon** € 34,45/ month (30mg = 1DDD)

A few studies are known with positive results on clinical endpoints. Has a slightly favorable effect on the lipid spectrum compared to rosiglitazone. Classified in step 2 of the NHG guideline.

**Rosiglitazon** € 44,30/ month (6mg = 1DDD)

Little information available on results at hard endpoints. If a TZD is prescribed: preferably choose Pioglitazone. Recently the potentially increased risk of fracture and coronary heart disease when using this drug is discussed. It is unclear how to deal with this in practice.

**Repaglinide** € 14,47/ month (4mg = 1DDD)

Functioning similar to those of sulfonylureas, however bind to other binding site, and has shorter working time. The efficacy is comparable to that of sulfonylureas and metformin. However no results are known on clinical endpoints. May be prescribed by impaired renal function.

**Acarbose** € 17,63/ month (0,3gr = 1DDD)

Alpha glucosidase inhibitor. Because of its many side effects and low blood glucose lowering capacity this drug should be considered obsolete.

**Combination preparations:**

The NHG guideline does not mention combination preparations.

Metformin-glibenclamide € 6,50/ month

Metformin-rosiglitazone € 34,73-58,31/ month

Glimepiride-rosiglitazone € 45,63-75,79/ month

**Insulin** 1DDD = 40E

Insulins are distinguished by duration of action:

- Short acting
- Medium long acting
- long acting
- mix insuline

The choice for a certain type of insulin is dependent of the treatment regimen that is followed (see below).

Step III: medium long acting insulin once daily to be administered next to oral drugs (stop TZD use). Start with 10^E^ after dinner. adjust dosage according to the diagram at the next page (figure 2) guided by the fasting glucose levels.

Step IVa: medium long acting or mix insulin twice daily. Start with 80% of the daily dose of step III. Divide according to: 66% prior to the breakfast and 33% prior to diner. Adjust dosage guided by fasting glucose levels and glucose levels after the meals. Continue the use of Metformin

Step IVb: Start with 80% of the daily dose of step III. Start with 3x 20% short acting insulin prior to the meals and 1x 40% long acting insulin before sleep

**Glucagon** € 24,81 (per ampoule) (1mg = 1 ampoule = 1DDD)

Blood glucose enhancer. Promotes glycogenolysis in the liver causing the blood sugar level to rise rapidly. Only works if there is stocked glycogen in the liver. Because of the small number of prescriptions and because it is not clear which part of the prescriptions are actually administered, you will get no feedback on this product.

14 E

18 E

20 E

2-3

days

2-3

days

2-3

days

2-3

days

10 E

Gluc 7-10

Gluc >10

Gluc >10

Gluc 4-7

fig 2. diagram for initiating insulin therapy (step III).

adjust the dose every 2-3 days guided by the

fasting glucose.

0

Fig 1. flowchart diabetes mellitus type 2 treatment. When targets are not reached advance to the next step.

*Source: NHG guideline M01, diabetes mellitus type 2. Pharmacotherapeutic compass 2007*

**Module Urinary tract infections**

Notions

Incidence: acute cystitis ♀ 70/ 1000 patients/ year

♂ 10/ 1000 patients/ year

pyelonephritis ♀ 2/ 1000 patients/ year

♂ <1/ 1000 patients/ year

prostatitis 2/ 1000 patients/ year

The incidence of acute cystitis in women has a peak between 15 and 24 years and after 60 years. In women 75 years of age, the incidence is 200/1000 patients / year.
In men, the peak incidence peaks after the age of 50. In men of 80 years the incidence is 80/1000 patients / year.

Cystitis is the most common complaint in women consulting a GP.

**Part A, diagnostics**

General:

The diagnosis urinary tract infection can only be determined in a patient with symptoms. Laboratory diagnostics is only valid as a complement, the history is the most important. Physical examination is recommended in patients at risk, with signs of tissue invasion and in women with recurrent urinary tract infections (single study).

Collection of urine: It is no longer preferred to make use of special measures for the reception of urine. Only when a discrepancy between symptoms and findings on examination exists it may be considered to catch the urine after spreading the labia or retract the prepuce.

Use preferably first morning urine. Maximum storage room temp: 2 hours, refrigerator: 24 hours.

Control after an uncomplicated cystitis is **not** necessary, provided the patient is free of symptoms.

**Nitrite test urine, POC** (text but no feedback) € 0,27 - 1,10 /stick

Based on the presence of reductase containing bacteria, they convert nitrate to nitrite. A positive nitrite test in case of complaints justifies treatment as cystitis. A negative nitrite test with complaints cannot reject the diagnosis of cystitis.
The NHG guideline recommends this test to be carried out first.
Control, in an asymptomatic patient, after an uncomplicated cystitis is not necessary. Reliability testing has not been studied in this situation.

**Leukocyte test urine, POC** (text but no feedback) € 0,27 - 1,10 /stick

Has as isolated test little impact on the confirmation or rejection of the diagnosis cystitis. As an addition to the nitrite test this test just seems important if both results are negative a cystitis is highly unlikely. Control in an asymptomatic patient after an uncomplicated cystitis is not necessary.

Reliability testing has not been studied in this situation.

**Hemoglobin test urine POC** (text but no feedback) € 0,27 - 1,10 /stick

This test provides no added value in addition to the tests above for the confirmation or rejection of the diagnosis cystitis. The NHG guideline does not recommends this test in the context of diagnosing cystitis. Control in an asymptomatic patient after an uncomplicated cystitis is not necessary.

Reliability testing has not been studied in this situation.

**Dip slide, POC** (text but no feedback) € 1,06 - 1,50 / piece

Can be used in the general practice in order to confirm or exclude a cystitis. Cut-off point is 104 bacteria per ml. Older studies indicate that the slide is a reliable test. A 2003 study questions this.

**Sediment, POC** (text but no feedback) € 1,41

Is to be used in a negative nitrite test to rule out the diagnosis cystitis. Only reliable if done by skilled investigator and the microscope is well maintained.

**Urine culture with antibiotic resistance testing** (price depends on lab)

First choice after two blind started therapies have failed and in complicated urinary tract infections. The urine must be collected before starting new antibiotic therapy.

If a culture shows a group B streptococcus (GBS) any pregnant woman should be referred to secondary care, regardless of gestational age and susceptibility. Recolonization with GBS occurs very frequently. Neonatal GBS infection has a high morbidity and mortality.

*Source: NHG guideline M05, urinary tract infections. NHG LESA test ordering rationally 2006. Diagnostic compass 2003*

**Flowchart UTI**

**Module urinary tract infections**

**Part B, pharmacotherapy**

General:

An uncomplicated urinary tract infection is treated for up to 5 days.
A complicated urinary tract infection is treated for 7-10 days.

Treat as complicated UTI when: - fever or tissue invasion is present

- in men

- in boys up to 12 years

- in girls up to 4 years

- in presence of anatomic anomalies to kidneys or urinary tract

- with an impaired defense (excl. diabetics)

- with a catheter in situ

**Nitrofurantoin** € 2,76 /week, **Furabid** € 3,73 /week (1 DDD = 0,2 g)

drug of first choice in an uncomplicated UTI. 2 times daily 100mg for 5 days (as long as Furabid® isn’t available: 4dd 50mg). Antibiotic resistance of E. Coli is very limited. A 7 day treatment regimen has the same effects as a 5 day regimen.

Contraindications: renal impairment, G6PD deficiency, just before / during childbirth.

Men, girls between 5 and 12 years, pregnant women and diabetics without tissue invasion: treat for 7 days.

**Trimethoprim** € 2,35 /week, € 1,64 /week (child, suspension) (1 DDD = 0,4 g)

Drug of second choice according to the NHG guideline. First choice with intolerance for nitrofurantoin. Once daily 300mg for 3 days.

note: local antibiotic resistance patterns can influence the choice of the preferred drug.

**Fosfomycin** € 5,98 /week (1 sachet) (1 DDD = 3 g)

Drug of third choice according to the NHG guideline. Prescribe 3 grams once. Start when nitrofurantoin and trimethoprim cannot be administered.

**Amoxicilline-clavulaanzuur** € 11,73 - 27,63 /week, € 2,34 - 8,28 /week (child) (1 DDD = 1 g)

Drug of first choice in complicated UTIs.

**Co-trimoxazol** € 2,55/week, € 1,17 - 1,30/week (child)

Drug of second choice in complicated UTIs. prescribe 2 times daily 960mg for 10 days. Not to be prescribed to pregnant women.

Note: local antibiotic resistance patterns can influence the choice of the preferred drug.

**Norfloxacine** € 5,64 - 5,96/week (1 DDD = 0,8 g)

Reserve antibiotic for urinary tract infections, only prescribe guided by antibiogram. Prescribe twice daily 400mg for 10 days.

**Ciprofloxacine** € 17,50 - 39,41/week (1 DDD = 1 g)

Reserve antibiotic for urinary tract infections, only prescribe guided by antibiogram. Prescribe twice daily 500mg for 10 days. Do not prescribe to pregnant women or youngsters <16 years.

*Source: NHG guideline M05, Urinary tract infections. Pharmacotherapeutic compass 2007*

**Module anaemia**

Notes

Anaemia: Low hemoglobin levels considering age and sex (according to normal reference range from regional laboratory)

Mild: Hb > 6,0 mmol/l (women and children) of > 6,5 mmol/l (men)

moderate: Hb > 5,0 mmol/l but < 6,0 mmol/l (women and children) or < 6,5

mmol/l (men)

severe: Hb < 5,0 mmol/l

Microcytic: mean corpuscular volume (MCV) <80 fl

Normocytic: 80 fl < MCV < 100 fl

Macrocytic: MCV > 100fl

**Part A, diagnostics**

Background:

Reference values are usually calculated based on a group healthy individuals, the 97,5 and 2.5 percentile are boundaries. Random blood tests performed in healthy thereby results in 5% abnormal results which does not indicate disease. The likelihood of an abnormal result increases as more tests are pending to a person. At 5 tests this chance is increased to 23%. testing Hb levels because of fatigue does not seem rational. The patient however can consider the "magic" of blood tests as satisfactory.

Risk factors for anaemia: º bloodloss

º abnormal diet (including alcohol abuse)

º infectious disease within last month (children)

º at risk for thalassemia (family history, immigrant descent)

º gastrointestinal malignancy

º old age

º chronic disease

Incidence: 6 per 1000 patients each year iron deficiency anaemia

0,5 per 1000 patients each year other deficiency anaemias (esp. vit. B_12_)

0,15 per 1000 patients each year other anaemia

General advice

Since iron deficiency anemia is the most common **simultaneous determination of Hb, MCV and ferritin is the most appropriate**.

**Hb/ Ht** € 11,08/ 1,41

The common Hb meters have a measurement error of 0.2 mmol / l. The Hb in capillary blood is on average 0.14 mmol / l lower than the value determined from venous blood. The milking of a finger can easily lead to a low value by mixing with tissue fluid. There is no difference in Hb of patients who complain of fatigue and patients who do not. Treatment does not lead to improvement of symptoms. Testing Ht is meaningful in cases of a slightly increased or slightly decreased Hb. This could be due to a dilution or thickening effect.

**Indices** € 1,41 a piece

Of the indices is just the MCV useful for the diagnosis of anemia in primary care. MCV helps classifying microcytic, normocytic or macrocytic anaemia. An increase in the number of reticulocytes (see there) can lead to an increase in the MCV because reticulocytes are larger than mature erythrocytes.

**Ferritin** € 8,31

Ferritin is a good indicator for the reserve-iron in the body, 1 μg / l of ferritin = 8μg reserve-iron. Ferritin is an acute phase protein and thus test results can be disrupted by infections.
Ferritin <15 μg / l indicates an iron deficiency anemia, further diagnosis of the iron status is not necessary. Values above 100 μg / L exclude an iron deficiency anemia.

**Folic acid** € 6,65

Folic acid is responsible for the synthesis of nucleic acid (DNA). Absorption takes place in the small intestine. Folate deficiency anaemia are rare in primary care.

**Vitamin B_12_** € 8,31

A macrocytic anaemia is usually caused by a vitamin B12 deficiency. The most common cause of vitamin B12 deficiency is the presence antibodies against intrinsic factor in the stomach (pernicious anemia).

**Reticulocytes** € 2,77

Determination only makes sense when further investigation is needed in the event of a macrocytic or normocytic anaemia.

Increases in the value indicative of hemolysis or blood loss and the exertion of the body to supplement the shortage of erythrocytes. A decrease indicates a shortage of nutrients (vitamin B12, folic acid or iron) or a production disorder. 4-5 days after onset of replacement therapy increases the value with a peak at day 7 (reticulocyte crisis).

**Serum iron** € 5,55

Useless in following up on iron deficiency.

Needs rarely be tested in primary care.

50% of the patients exhibit a circadian difference in serum iron content of 30%. Serum iron therefore should tested preferably in the morning (fasting). In over or improper supplementation the serum iron will be elevated with normal or reduced transferrin levels.

**Bilirubin** € 1,41

Has no place in the diagnosis of anemia in the first line. Not useful when diagnosing hemolysis.

**LDH** € 1,41

Is rarely needed, vitamin B12 and folic acid levels provide sufficient information. LDH is present in every cell of the body. Damage to the cell leads to release of LDH. In the case of a haemolytic anaemia the LDH is increased up to 3 times the normal value. In the case of an untreated megaloblastic anaemia due to deficiency of folic acid LDH levels will have increased more than 3-fold.

**Transferrin/ Total iron binding capacity (TIJBC)/ transferrin saturation**  € 5,55

Transferrin: hardly ever indicated in primary care considering anaemia diagnostics.

TIJBC: complete iron binding capacity of transferrin together with a small contribution of albumin. Is considered to be obsolete.

Transferrin saturation: hardly ever indicated in primary. In repeated tests >60%: higher probability of hemochromatosis.

*Source: NHG guideline M76, anaemia. NHG LESA test ordering rationally 2006. Diagnostic compass 2003*

**Algorithm anaemia for GPs**

result

test

result

test

result

Diagnosis

This algorithm provides a pathway to interpret the most common combinations of test results; Less common combinations of results are more difficult to interpret.

Hb: Hemoglobin N: normal value ACD: anaemia caused by chronic disease

LDH: lactate dehydrogenase MCV: mean corpuscular volume : most frequent diagnoses

**Module anaemia**

**Part B, Pharmacotherapy**

General

The following drugs serve as supplementation for an existing shortage of the substance. The cause of a deficiencyshould be treated when possible. This is is beyond the scope of this summary. Doses higher than 100mg ferrous iron per day result in more side effects. Administration with calcium can inhibit the absorption of ferrous iron.

**Ferrous sulfate (= slow release)** € 4,40/ month (1 DDD = 105mg Fe^++^)

Not reccomended.

Because iron is absorbed almost exclusively in the first part of the intestine, slow release preparations lead to a lower absorption of iron and therefore have a lower impact on the hemoglobin content of the blood. The biological availability of divalent iron compounds amounts to 46 to 100%; for slow-release formulations it is at 31-47%.

**Ferrous fumarate** € 4,38/ month (tabl), € 13,17/ month (susp), € 5,34/ month (child) (1 DDD = 195mg Fe^++^)

Drug of first choice according to the NHG guideline. Divalent iron has a good absorption and bioavailability. 3 times daily 200mg or 2-4mg / kg ferrous as suspension (child) distributed over three doses. The suspension can give tooth discoloration, intake with a straw prevents this.

**Ferrous gluconate** € 15,28/ month (1 DDD = 154mg Fe^++^), (tablet: 1 DDD = 80mg Fe^++^)

The suspension is drug of second choice according to the NHG guideline. Adults: 3 times daily 6ml; children 2-4mg ferro/kg distributed over 3 doses. The suspension can give tooth discoloration, intake with a straw prevents this.

The effervescent tablet is not included in the standard. It can give tooth discoloration. The claim that there are fewer gastrointestinal side effects in effervescent tablets is not or not sufficiently supported by evidence.

**Ferrous chloride** € 4,11/ month, € 1,23/ month (child, susp) (1 DDD = 150mg Fe^++^)

Not mentioned in the NHG guideline. The suspension can give tooth discoloration, intake with a straw prevents this.

**Folic acid (vit B_11_)** € 0,76 /month (based on 5 mg tablets!) (1 DDD = 0,4 mg)

In proven folic acid deficiency folic acid can be prescribed with 0.5mg once daily. After 6-12 weeks the administration can be discontinued. The cause of the deficiency needs to be treated if possible. Green leafy vegetables are rich in folic acid.
Supplementation without pernicious anaemia ruled out seems unwise because of increased risk of neurological abnormalities. Most times folic acid and vitamin B12 are tested together.

Note: Most folic acid tablets are freely available without prescription, they are not included in the figures as presented.

**Vitamine B_12_** € 0,61/ ampoule 1 ampoule = 50DDD’s

In a proven vitamin B12 deficiency the GP starts with 10 injections of 1mg hydroxocobalamin with an interval of 3 days. After this, one injection every two months. In case of a pernicious anaemia: treat lifelong.
Oral therapy does not appear necessary, by the lack of intrinsic factor is the added oral vitamin is not absorbed.
Hydroxocobalamin is the most natural and thus has the greatest bioavailability, hydrocobamine should not be prescribed for this reason.

*Source: NHG guideline M76, anaemia. Pharmacotherapeutic compass 2006.*

**Module stomach complaints**

Backgrounds

Prevalence of Helicobacter Pylori infections is much higher in immigrants due to endemic prevalence in the country of origin.

Incidence: 25 per 1000 stomach ache

7 per 1000 heartburn

14 per 1000 nausea

Diagnosis: 60-70% functional complaints or cause unknown, prognosis is poor, 50 to 90% has long term complaints.

20-25% gastroesophageal reflux

5% ulcus ventriculi, duodenal ulcer

< 1% malignancies

**Part A, diagnostics**

General

Alarm Symptoms: haematemesis, melaena, dyspepsia with persistent vomiting, passage disorders, unintentional weight loss and anaemia.
Risk factors for ulcer disease: previous ulcer, male sex, older age, smoking and hunger pains.
In the elderly one should be aware for in serious pathology in an earlier stage.
There are no reliable data on testing for H. pylori routinely or in persistent symptoms after eradication therapy. CBO guideline: only if the doctor can order a breath test after eradication, control is recommended at least 30 days after eradication.

**^13^C-urea breathtest/ ^14^C-urea breathtest** € 53,00/ € 121,00

Test of first choice according tot he NHG guideline (^13^C- urea breathtest). Sensitivity of the ^13^C method is >98%, specificity is >97%. The ^14^C method needs the presence of a nuclear medicine department in the referral hospital. The ^13^C method is used by the clinical chemistry department.

Treatment with PPIs or H_2_ receptor antagonists should be stopped 14 days prior to testing.

**HP- antibodies/ fecestest** € 12,28-18,00/ € 21,85

Some laboratories won’t perform H. Pylori antibody in serum testing anymore. Antibodies in serum are detectable up to 12 months after eradication.

De fecestest is only validated for use in secondary care. Both are unsuitable for testing after eradication therapy. Sensitivity and specificity of the antibody test are both 80 to >95%. Sensitivity of the fecestest is 80 to 90%, specificity >90%. The patient should not use PPIs 2 weeks prior to the test and no antibiotics 4 weeks prior to the test.

**Esophagoscopy/ gastroscopy** € 151,20 – 198,45/ € 352,67

Indications for endoscopy:

- If alarm symptoms, the patient is referred for endoscopy or a specialist.
- In elderly people with no history of stomach problems an endoscopy may be considered earlier.
- H. pylori negative patients who do not respond to treatment with acid inhibitors.
- No reduction of symptoms after eradication therapy (if no urea breath test is available).
- Esophagitis grade C and D after 12 weeks of treatment.
Sensitivity and specificity: depending on specialist performing endoscopy and medical history.
In GERD with good response to initial therapy, there is no indication for routine endoscopy.

**X-contrast stomach/ esophagus** € 114,24

There is hardly any indication for this type of X-ray. Endoscopy has greater diagnostic value and abnormalities seen on a photograph leads to an indication for endoscopy. Small differences are not seen on an X-stomach or esophagus.
In great fear of endoscopy a contrast image can be considered to detect gross abnormalities.

*Source: NHG guideline M36 stomach complaints. NHG LESA test ordering rationally 2006. Diagnostic compass 2003. Multidisciplinary guideline stomach complaints, CBO 2004.*

**Module stomach complaints**

**Part B, pharmacotherapy**

General

Start with antacids or H2 receptor antagonists. In many cases this approach is sufficient. After a year 75% of the patients had no symptoms anymore. The placebo effect is substantial.
The discussion of antiemetic and prokinetic drugs are beyond the scope of this module.
A distinction is made between a first episode or the last episode of > 1 year ago (without clear cause diagnosed) on the one hand and persistent or recurrent symptoms on the other.

**Antacids**

First choice at first episode of mild stomach complaints. Alghedrate / magnesiumoxide susp 4dd15ml one hour postprandial and before bedtime. Always subscribe for 2-4 weeks and then evaluate. Try to stop after eight weeks even in mild residual symptoms.

**H_2_- receptor antagonists** € 11,62 - 36,86 / month

First choice at first episode more severe stomach upset and typical reflux symptoms. Always subscribe for 2-4 weeks and then evaluate. Try to stop after eight weeks even in mild residual symptoms. After discontinuation rebound symptoms may occur after 3 weeks. Use in some cases a step-down scheme and sustain with antacids. The degree of rebound is positively correlated with the degree of acid inhibition.
Chronic use: after gastric bleeding or perforation after consultation with the treating specialist. In grade C or D chronic esophagitis continuous use is indicated. In all other cases a complaint-driven treatment is indicated.
Well useable in double dosage for the prevention of NSAID induced ulcers. More expensive than misoprostol.

Famotidine (1DDD=40mg), ranitidine (1DDD=0,3g), Cimetidine (1DDD=0,8g), Nizatidine (1DDD=0,3g)

**Protonpump inhibitors** € 8,83 – 39,37 / month

Drug of first choice for H. Pylori eradication, highly dosed.
Relative second choice (NHG: to begin with H2-receptor antagonists) in persistent or recurrent symptoms. Try-out treatment for 2-4 weeks. When results are satisfying: continue up to 8 weeks. Hereinafter administer intermittent (2-4 weeks) or on demand. Evaluate biannually for instance its use and the complaints. 6-14 days after discontinuation might a rebound effect occur. The degree of rebound is positively correlated with the degree of acid inhibition. If a patient H. Pylori is positive then this should be treated as well.
Chronic use: after gastric bleeding or perforation after consultation with the treating specialist. In grade C or D (≈ III-IV) chronic esophagitis continuous use is indicated. In all other cases, a complaint-driven treatment is indicated.
Useful for the prevention of NSAID-induced ulcers. More expensive than misoprostol.

Omeprazole (1DDD=20mg), Pantoprazole (1DDD=40mg), Lansoprazole (1DDD=30mg), Rabeprazole (1DDD=20mg), Esomeprazole (1DDD=30mg)

**Claritromycine** € 8,71 – 10,30 / week, € 3,01 – 6,03 /week (kind) (1 DDD = 0,5 g)

Claritromycin 2 times daily 500 mg in combination with amoxicillin and a proton pump inhibitor (triple therapy) for 7 days is an effective eradication therapy. Locally based on abnormal resistance patterns different combination of antibiotics or other dosages can be preferred.

**Misoprostol** € 31,25 / month (1 DDD = 0,8 mg)

prostaglandin E1 analogue with both mucosa protective and gastric acid inhibiting effect. Each dosage can cause diarrhea. The degree of side effects depends on the dose. If tolerated well by the patient it is useful for the prevention of NSAID-induced ulcer disease and in addition it is cheap.

**Pantopac** € 275,71 / month

No judgment rendered by NHG. Contains triple therapy in dose as recommended by NHG.

Note the NHG guideline mentions no proton pump inhibitor of choice.

**Amoxicilline** € 4,75 - 4,77 / week, € 1,23 – 2,28 / week (child) (1 DDD = 1 g)

2 times daily amoxicillin 1000 mg in combination with clarithromycin and a proton pump inhibitor (triple therapy) for 7 days is an effective eradication therapy. Locally based on abnormal resistance patterns different combination of antibiotics or other dosages can be preferred. In penicillin allergy to be replaced by metronidazole.

**Tetracycline** € 1,05 / week (1 DDD = 1 g)

Part of quadruple therapy, apply if triple therapy proven to have failed. Dosage 4 times daily 500 mg, 7 days. Combining with proton pump inhibitor, dosed high. The quadruple therapy is the second choice, despite the limited influence of resistance patterns because of the complexity of the treatment and the related lower compliance.

**Metronidazol** € 3,80/week (1 DDD = 2 g)

Part of quadruple therapy, apply if triple therapy proven to have failed. Dosage 3 times daily 500 mg, 7 days. Combining with proton pump inhibitor, dosed high. The quadruple therapy is the second choice, despite the limited influence of resistance patterns because of the complexity of the treatment and the related lower compliance.

*Source: NHG guideline M36 stomach complaints. Pharmacotherapeutic compass. Multidisciplinary guideline stomach complaints, CBO 2004.*

**Flowchart stomach complaints**

**stomach complaints**

first episode o last episode > 1 year ago without a clear cause

Persistent or recurring

Alarm symptoms

endoscopy

Diagnostic certainty desirable

H. Pylori antibodies o fecestest

Try-out treatment with PPIs

Education and advice; antacids or H_2_ receptor antagonists

Typical GERD complaints

Other complaints

*Source: NHG guideline M36 stomach complaints.*

**Module Thyroid dysfunction**

Notes:

Hypo and hyperthyroidism: clinical condition characterized by typical complaints proven by testing TSH and FT4 levels.

hypothyroidism: TSH ↑ and FT4 ↓

hyperthyroidism: TSH ↓ and FT4 ↑

subclinical hypothyroidism: state in which the TSH deviates and FT4 levels are normal

Myxedema: pasty swelling of the skin, non-pitting. Occurs in
both hypo- and hyperthyroidism. Hyper: especially pretibial, hypo: especially around the eyes and rest of face.

Thyroid nodules: localized enlargement of the thyroid gland

Struma: enlargement of the thyroid gland diffuse of several
nodules (Multi-nodular).

M. Hashimoto: chronic autoimmune thyroiditis. mainly in
women. Non-iatrogenic hypothyroidism: 90-95% Hashimoto.

M. Graves autoimmune thyroiditis TSI (see below) attaches itself to
receptors like TSH does.

silent thyroiditis: autoimmune thyroiditis with destruction and regeneration
thyroid tissue recovery in 1-4 months.

Postpartum thyroiditis: see under silent thyroiditis occurs in 7% of women in the first year postpartum. 25-50% chance of the occurrence of a long-term hypothyroidism.

Incidence primary care: hypothyroidism: 1,2/ 1000 a year (increased incidence with Down syndrome)

Hyperthyroidism: 0,5/ 1000 a year

Malignancies: 2,8/ 100.000 a year(♀) en 1,3/ 100.000 a year (♂)

Prevalence primary care: thyroid nodules: 0,4/ 1000

Struma: 0,7/ 1000

**Part A, diagnostics**

General advice

Always determine upon suspicion of thyroid dysfunction the presence TSH and FT4 together (if not included in the cascade on the problem-based test ordering form).
Always determine when M. Graves is suspected the TSI (besides TSH and FT4).
Pursue an euthyroid situation in women who are pregnant but have had a (subclinical) hypothyroidism or hyperthyroidism in the past. Thyroid abnormalities impair cerebral development of the child in utero. Consult a qualified specialist internal medicine or gynecologist about referral.

General

The thyroid gland produces 4x more T4 than T3. T4 is metabolized into the biological active T3 in peripheral tissue.

95% of all thyroid dysfunction is primary (reason within the thyroid gland itself). 5% of the thyroid dysfunction is secondary (caused by pituitary gland dysfunctioning or CNS disease).

**TSH** € 5,55

Thyroid stimulating hormone

Determination of TSH is the best screening test for thyroid problems. A normal TSH virtually excludes thyroid dysfunction.
TSH can be determined if a patient has symptoms consistent with a hypothyroidism or hyperthyroidism. There is no pathognomonic symptom pattern (excl. Ophtalmopathy).

Increased risk of thyroid dysfunction in:

- Treated hyperthyroidism
- history of radiotherapy of head and neck
- Recent parturition (<1 year ago) or history of hypothyroidism postpartum
- Presence of other autoimmune disease (particularly DM type I)
- Down’s syndrome
- Positive family history
- Use of iodine-containing drugs (lithium, amiodarone)
- Status after hypovolemic shock; due to necrosis or atrophy of the pituitary gland (trauma, post-partum haemorrhage).

Follow-up: after hyperthyroidism 1 Year after discontinuation of medication. A renewed low TSH means there is a relapsing disease.

**FT4** € 6,65

Free T4, that part of the total thyroxine in serum that is bound to protein.
Is lower in the third trimester of pregnancy.
Is typically automatically determined by the laboratory at an abnormal TSH level. Is also used to monitor the treatment of hyperthyroidism and adjust the therapy.

**T3** € 8,31

tri-iodo thyronine, biologically active thyroid hormone, in particular found in tissues outside the thyroid gland. Provides a better reflection of the biological effect than FT4 or T4. should not be requested as a separate test. Indication: Excluding T3 toxicosis.

**TSI** € 8,31

TSH-receptor stimulating antibodies, stimulating the thyroid gland. Presence indicates Graves' disease.
Determine when Graves' disease is suspected and in pregnant women who have had hypothyroidism in the past.

**TPO-Ab** € 8,31

antibodies mainly found in Hashimoto's thyroiditis, the antibodies destroy the thyroid. In other thyroid disorders slightly increased values can be detected.

**ultrasound thyroid gland** € 56,52

Does not contribute to the diagnosis of thyroid dysfunction. Meaningful in the context of diagnosis of palpable abnormalities of the thyroid gland which are not accompanied by thyroid dysfunction.

Determination of ESR, leukocytes and differentiation for diagnosing thyroid dysfunction are so unspecific that no feedback can be provided.

Requesting a nuclear scan of the thyroid gland is for most GPs not possible. Therefore you do not receive feedback on this item.

*Source: NHG guideline M31, Thyroid diseases. NHG LESA test ordering rationally 2006. Diagnostic compass 2003.Memobook for primary care diagnostics, SAN 2007.*

**Module schildklierstoornissen**

**Part B, Pharmacotherapy**

General

In the case of treatment of hypothyroidism following applies: "start low, go slow". Ensure that fertile women in your population with a known hypothyroidism contact you if they want to be pregnant or may be pregnant. The need for thyroxine rises in pregnancy. The need is lower in the elderly.
Check treatment of hypothyroidism every 6 weeks (TSH) until levels are appropriate, earlier does not make sense.
Pregnancy: Check every 4 weeks. Optional: start "blind" with twice every week an additional daily dose from the start of the pregnancy.

**Levothyroxine sodium** € 1,25 - 1,79 / month (1 DDD = 0,15 mg)

Substitution therapy for hypothyroidism, drug of first choice. Average dose in total failure of the thyroid gland is 1.6 micrograms / kg / day. Adjust the dose guided by the TSH and complaints.

**Liothyronine sodium**  € 40,36 / month (1 DDD = 0,60 mg)

Combined with levothyroxine as combination therapy offers no advantage over monotherapy with levothyroxine. Same mechanism of action as levothyroxine, it takes effect a few days earlier and holds a few days less. Is not included in the NHG guideline as monotherapy.

**Thiamazole** € 1,36 / month (1 DDD = 10 mg)

Titration method: increase the dose guided by the FT4 (eg. in pregnant women.)
Combination method: once daily 30mg, it stops the thyroid gland. Add-back therapy with levothyroxine guided on the FT4. determination of TSH is not necessary. When M Graves: stop all medication after 1 year and follow thyroid function. When (multi) struma: lifelong treatment.
Note: therapy requires special interest and knowledge.

**Propylthiouracil** (no feedback due to low volumes)

Antithyroid drug not included in NHG guideline.

**Carbimazole** (no feedback due to low volumes)

Antithyroid drug not included in NHG guideline.

*Source: NHG guideline M31, Thyroid diseases. Pharmacotherapeutic compass 2007.*
