# Supplementary material for: Effect of audit and feedback with peer review on general practitioners’ prescribing and test ordering performance: a cluster-randomized controlled trial
Source: BMC Fam Pract. 2017 Apr 13;18:53. doi: 10.1186/s12875-017-0605-5 (PMC5390393; doi:10.1186/s12875-017-0605-5)
Supplement: Supplementary file 2 — Complete set of drugs and tests included in the databases for this intervention. (DOCX 33 kb) [file 12875_2017_605_MOESM2_ESM.docx]

**Appendix 2**

Complete set of drugs and tests included in the databases for this intervention

Appendix for: Effect of audit and feedback with peer review in existing infrastructure in the south of the Netherlands on prescribing and test ordering performance of general practitioners after implementation: A cluster randomized controlled trial.[ISRCTN40008171]

J. Trietsch, B. v. Steenkiste, R. Grol, B. Winkens, H. Ulenkate, J. Metsemakers, T. van der Weijden

| **Module** | **ATC** | **Drug** | **label on FB form** |
| --- | --- | --- | --- |
| **Diabetes mellitus 2** | A10BA02 | metformin | metformin |
|  | A10BB09 | glicazide | glicazide |
|  | A10BB12 | glimepiride | glimepiride |
|  | A10BB03 | tolbutamide | tolbutamide |
|  | A10BB01 | glibenclamide | glibenclamide |
|  | A10BG02 | rosglitazone | rosiglitazone |
|  | A10BG03 | pioglitazone | pioglitazone |
|  | A10BX02 | repaglinide | other oral antidiabetics |
|  | A10BF01 | acarbose | other oral antidiabetics |
|  | A10BH01 | sitagliptin | DPP4 inhibitors |
|  | A10BH02 | vildagliptin | DPP4 inhibitors |
|  | A10BH03 | saxagliptin | DPP4 inhibitors |
|  | A10BX04 | exenatide | incretines |
|  | A10BX07 | liraglutide | incretines |
|  | A10BD04 | glimepiride/ rosiglitazone | combination preparations |
|  | A10BD02 | metformin/ glibenclamide | combination preparations |
|  | A10BD05 | pioglitazone/ metformin | combination preparations |
|  | A10BD03 | metformin/ rosiglitazone | combination preparations |
|  | A10BD07 | sitagliptin/ metformin | combination preparations |
|  | A10BD08 | vildagliptin/ metformin | combination preparations |
|  | A10AB | insuline short acting | insuline short |
|  | A10AC | insuline medium long acting | insuline medium long |
|  | A10AD | insuline mix | insuline mix |
|  | A10AE | insuline long acting | insuline long |
|  |  |  |  |
| **stomach complaints** | A02AA | Magnesium compounds | antacids |
|  | A02AB | Aluminium compounds | antacids |
|  | A02AD | combinations and complexes of al- ca- en mg-compounds | antacids |
|  | A02AH | Antacids with sodium bicarbonate | antacids |
|  | A02BA03 | famotidine | other H2-antagonists |
|  | A02BA02 | ranitidine | ranitidine |
|  | A02BA01 | Cimetidine | other H2-antagonists |
|  | A02BA04 | Nizatidine | other H2-antagonists |
|  | A02BC01 | Omeprazole | omeprazole |
|  | A02BC02 | Pantoprazole | pantoprazole |
|  | A02BC03 | Lansoprazole | lansoprazole |
|  | A02BC04 | Rabeprazole | rabeprazole |
|  | A02BC05 | Esomeprazole | esomesoprazole |
|  | A02BB01 | Misoprostol | misoprostol |
|  | A02BD04 | pantoprazole, amoxicillin and clarithromycin, fixed combination | pantopac |
|  |  |  |  |
| **Anaemia** | B03AA07 | ferrous sulfate | ferrous sulfate |
|  | B03AA02 | ferrous fumarate | ferrous fumarate |
|  | B03AA03 | ferrous gluconate | ferrous gluconate |
|  | B03AA05 | ferrous chloride | ferrous chloride |
|  | B03BA01 | vitamin B12 | vitamin B12 |
|  | B03BA03 | vitamin B12 | vitamin B12 |
|  | B03BB01 | folic acid | folic acid |
|  |  |  |  |
| **Chlamydia infections** | J01AA02 | Doxycycline | doxycycline |
|  | J01FA10 | Azithromycin | azithromycin |
|  | J01CA04 | Amoxicillin | amoxicillin |
|  | J01FA01 | Erythromycin | erythromycin |
|  |  |  |  |
| **Prostate complaints** | G04CA01 | Alfuzosin | alfuzosin |
|  | G04CA02 | Tamsulosin | tamsulosin |
|  | C02CA04 | Doxazosin | other α1 blockers |
|  | G04CA03 | Terazosin | other α1 blockers |
|  | C02CA01 | Prazosin | other α1 blockers |
|  | G04CB01 | Finasteride | finasteride |
|  | G04CB02 | Dutasteride | dutasteride |
|  |  |  |  |
| **Rheumatic complaints** | M01AA01 | phenylbutazone | other NSAIDs |
|  | M01AB01 | Indometacin | indometacin |
|  | M01AB05 | Diclofenac | diclofenac |
|  | M01AB02 | Sulindac | other NSAIDs |
|  | M01AB16 | Aceclofenac | other NSAIDs |
|  | M01AC06 | meloxicam | preferential cox-2 inhibitors |
|  | M01AC01 | piroxicam | preferential cox-2 inhibitors |
|  | M01AC02 | tenoxicam | other NSAIDs |
|  | M01AE01 | ibuprofen | ibuprofen |
|  | M01AE02 | naproxen | naproxen |
|  | M01AE03 | ketoprofen | other NSAIDs |
|  | M01AE09 | flurbiprofen | other NSAIDs |
|  | M01AE11 | tiaprofenic acid | other NSAIDs |
|  | M01AE14 | dexibuprofen | other NSAIDs |
|  | M01AE17 | dexketoprofen | other NSAIDs |
|  | M01AG02 | tolfenamic acid | other NSAIDs |
|  | M01AX04 | azapropazone | other NSAIDs |
|  | N02BA11 | diflusinal | other NSAIDs |
|  | N02BB02 | metamizole sodium | other NSAIDs |
|  | M01AH01 | celecoxib | coxibs |
|  | M01AH05 | etoricoxib | coxibs |
|  | M01AX01 | nabumetone | preferential cox-2 inhibitors |
|  | M01AB55 | diclofenac+ misoprostol | diclofenac+ misoprostol |
|  | A07EC01 | Sulfasalazine (tekst but no feedback) | DMARD's |
|  | L01BA01 | Methotrexate (tekst but no feedback) | DMARD's |
|  | L04AA13 | Leflunomide (tekst but no feedback) | DMARD's |
|  | L04AA11 | Etanercept (tekst but no feedback) | DMARD's |
|  | L04AA12 | Infliximab (tekst but no feedback) | DMARD's |
|  | M01CB | gold preparations (tekst but no feedback) | DMARD's |
|  | M01CC01 | d-penicillamine (tekst but no feedback) | DMARD's |
|  | P01BA01 | chloroquine (tekst but no feedback) | DMARD's |
|  | L04AX01 | azathioprine (tekst but no feedback) | DMARD's |
|  | L04AD01 | ciclosporin (tekst but no feedback) | DMARD's |
|  |  |  |  |
| **Thyroid dysfunction** | H03AA01 | Levothyroxine sodium | Levothyroxine |
|  | H03AA02 | Liothyronine sodium | Liothyronine |
|  | H03BB02 | Thiamazole | Thiamazole |
|  |  |  |  |
| **Urinary tract infections** | J01XE01 | Nitrofurantoin | Nitrofurantoin |
|  | J01EA01 | Trimethoprim | Trimethoprim |
|  | J01XX01 | Fosfomycin | Fosfomycin |
|  | J01CR02 | Amoxicillin with enzyme inhibitor | Amoxicilline-clavulaanzuur |
|  | J01EE01 | sulfamethoxazole with trimethoprim | Co-trimoxazol |
|  | J01MA06 | Norfloxacin | Norfloxacin |
|  | J01MA02 | ciprofloxacin | ciprofloxacin |
|  |  |  |  |
| **Dyslipaedemia** | C10AA01 | Simvastatin | simvastatin |
|  | C10AA03 | Pravastatin | pravastatin |
|  | C10AA05 | Atorvastatin | atorvastatin |
|  | C10AA04 | Fluvastatin | fluvastatin |
|  | C10AA07 | Rosuvastatin | rosuvastatin |
|  | C10AB02 | Bezafibrate | fibrates |
|  | C10AB04 | Gemfibrozil | fibrates |
|  | C10AB08 | Ciprofibrate | fibrates |
|  | C10AD06 | Acipimox | nicotic acid (analogous) |
|  | C10AX09 | ezetimibe | ezetimibe |
|  | C04AD02 | xantinol nicotinate | nicotic acid (analogous) |
|  | C10AD02 | nicotic acid | nicotic acid (analogous) |
|  | C10AC01 | Colestyramine | colestyramine |
|  | C10AX | ezetimibe/ simvastatin | combination preparate |
|  |  |  |  |
| **perimenopausal complaints** | G03CA03 | estradiol | estrogen monotherapy |
|  | G03CA04 | estriol | estrogen monotherapy |
|  | G03CA01 | ethinylestradiol | estrogen monotherapy |
|  | G03CA57 | geconjugated estrogens | estrogen monotherapy |
|  | G03AA | progestogens and estrogens, fixed combinations | contraceptives, >50 jaar |
|  | G03AB | progestogens and estrogens, fixed combinations | contraceptives, >50 jaar |
|  | G03AC | progestogens and estrogens, fixed combinations | contraceptives, >50 jaar |
|  | G03BB01 | progestogens and estrogens, fixed combinations | contraceptives, >50 jaar |
|  | G03HB01 | estradiol/cyproterone (Climene '28' ) | combination preparates |
|  | G03FA17 | estradiol/drospirenone (Angeliq) | combination preparates |
|  | G03FB08 | estradiol/dydrogesterone (Femoston) | combination preparates |
|  | G03FA14 | estradiol/dydrogesterone continuous (Femoston continu ) | combination preparates |
|  | G03FA01 | estradiol/norethisterone continuous (Activelle, Kliogest) | combination preparates |
|  | G03FB05 | estradiol/norethisterone (Estracomb TTS, Trisequens) | combination preparates |
|  | C02AC01 | clonidine | clonidine |
|  | G03DC05 | tibolone | tibolone |

| **Module** | **Item** | **codeNr** | **description labcode table** | **Mnemonic** | **Mat** | **Group** | **label on FB form** |
| --- | --- | --- | --- | --- | --- | --- | --- |
| **Diabetes mellitus 2** | Glucose random | 370 | Glucose time | GLUC | B | KC | glucose |
|  | Glucose not sober | 371 | Glucose not sober | GLUC | B | KC | glucose |
|  | Glucose sober | 372 | Glucose sober | GLUC | B | KC | glucose |
|  | HbA1c | 368 | glycohemoglobine (HbA1c) | GLHB | B | KC | HbA1c |
|  | Total cholesterol | 192 | cholesterol total | CHOL | B | KC | T-cholesterol |
|  | HDL | 446 | HDL-cholesterol | HDL | B | KC | HDL |
|  | LDL | 542 | LDL-cholesterol | LDL | B | KC | LDL |
|  | triglycerides | 1377 | triglycerides | TRIG | B | KC | triglyc |
|  | creatinine | 523 | creatinine | KREA | B | KC | creat |
|  | Albumin concentration, urine | 38 | albumin (micro-) urine portion | ALB | U | KC | albumin urine |
|  |  |  |  |  |  |  |  |
| **stomach complaints** | ^13^C-urea breathtest | 2130 | 13C urea breathtest | UREU | RU | FO | breathtest |
|  | ^14^C-urea breathtest |  | 14C urea breathtest |  |  |  | breathtest |
|  | Fecestest HP | 2184 | Helicobacter pylori ag. feces | HPYA | DF | IM | fecestest HP |
|  | Fecestest HP | 2107 | Helicobacter pylori ag. feces (PCR) | HPYP | DF | IM | fecestest HP |
|  | antibodies helicobacter pylori, IgG | 478 | Helicobacter pylori ab. IgG | HPYG | B | IM | antibodies HP |
|  | antibodies helicobacter pylori, general | 479 | Helicobacter pylori ab. | HPYI | B | IM | antibodies HP |
|  | antibodies helicobacter pylori, IgM | 480 | Helicobacter pylori ab. IgM | HPYM | B | IM | antibodies HP |
|  | gastroscopy | 2359 | gastroscopy | SCOP | DU | BO | gastroscopy |
|  | X-contrast stomach | 2333 | X-stomach | X | DU | BO | X-stomach |
|  |  |  |  |  |  |  |  |
| **Anaemia** | Hb | 412 | hemoglobin (Hb) | HB | B | HE | Hb/ Ht |
|  | Ht | 484 | hematocrit (Ht) | HT | B | HE | Hb/ Ht |
|  | anaemia diagnostics (cascade) | 2190 | anaemia diagnostics (Hb+MCV+flow chart) | ANEM | B | KC | anaemia cascade |
|  | Ferritin | 328 | ferritin | FERR | B | KC | ferritin |
|  | Bilirubin | 111 | bilirubin total | BILI | B | KC | bilirubin |
|  | folic acid | 336 | folic acid | FOLI | B | KC | folic acid |
|  | Vitamin B_12_ | 1412 | vitamin B12 | VB12 | B | KC | vit B_12_ |
|  | Reticulocytes | 1276 | Reticulocytes | RETI | B | HE | Reticulocytes |
|  | Serum iron | 323 | iron | FE | B | KC | Serum iron |
|  | LDH | 534 | lactaat dehydrogenase (LDH) | LDH | B | KC | LDH |
|  | total iron binding capacity | 1394 | iron binding capacity total | TYBC | B | KC | transerrin/ TIJBC |
|  | Transferrin | 1376 | transferrin | TRFE | B | KC | transerrin/ TIJBC |
|  | indices MCV | 582 | MCV | MCV | B | HE | MCV |
|  |  |  |  |  |  |  |  |
| **Chlamydia infections** | chlamydia DNAtest, urine (PCR) | 176 | Chlamydia DNA urine | CHDN | U | IM | chlamydia urine |
|  | chlamydia DNAtest, endocervical (PCR) | 177 | Chlamydia DNA urethra | CHDN | UU | IM | chlamydia endocervical/ vaginal |
|  | chlamydia DNAtest, vaginal swab (PCR) | 178 | Chlamydia DNA cervix | CHDN | XC | IM | chlamydia endocervical/ vaginal |
|  | antibodies chlamydia trachomatis, IgA | 182 | Chlamydia ab. IgA | CHIA | B | IM | antibodies CT |
|  | antibodies chlamydia trachomatis, IgG | 185 | Chlamydia ab. IgG | CHLG | B | IM | antibodies CT |
|  | antibodies chlamydia trachomatis, general | 186 | Chlamydia ab. | CHLI | B | IM | antibodies CT |
|  | antibodies chlamydia trachomatis, IgM | 191 | Chlamydia ab. IgM | CHLM | B | IM | antibodies CT |
|  |  |  |  |  |  |  |  |
| **Prostate complaints** | creatinine | 523 | creatinine | KREA | B | KC | creatinine |
|  | PSA | 896 | Prostate spec. ag. (PSA) | PSA | B | KC | PSA |
|  | PSA complex | 1921 | Prostate spec. ag. complex (PSA complex) | PSAC | B | KC | PSA |
|  | Free/Total PSA-ratio | 2124 | Free/Total PSA-ratio | PSAR | B | KC | V/T-PSA |
|  |  |  |  |  |  |  |  |
| **Rheumatic complaints** | Waaler-Rose | 1279 | Rheumatoid factors, Waaler-Rose | RFWR | B | IM | (semi-)qualitative rheumatoid factors |
|  | Latex fixation test | 2154 | Rheumatoid factor, Latex fixation | RFLF | B | IM | (semi-)qualitative rheumatoid factors |
|  | IgM-rheumafactor | 1278 | Rheumatoid factor quantitative | RF | B | IM | IgM rheumatoid factor |
|  | anti-cyclic citrullin peptide antibodies | 2153 | Rheumatoid factor (IgM ELISA) | RFIM | B | IM | anti-CCP |
|  | ESR | 134 | sedimentation (ESR) | BSE | B | HE | ESR |
|  | CRP | 227 | C-reactive protein (CRP) | CRP | B | IM | CRP |
|  | X-wrist left | 2239 | xwrist le | X | LO | BO | X-hand/ wrist |
|  | X-wrist right | 2240 | xwrist ri | X | LO | BO | X-hand/ wrist |
|  | X-hand left | 2243 | xhand le | X | LM | BO | X-hand/ wrist |
|  | X-hand right | 2244 | xhand ri | X | LM | BO | X-hand/ wrist |
|  | antistreptolysin titer | 81 | antistreptolysin titer (AST) | AST | B | IM | AST |
|  |  |  |  |  |  |  |  |
| **thyroid dysfunction** | TSH | 1385 | thyroid stimulating hormone (TSH) | TSH | B | KC | TSH |
|  | thyroid diagnostics (cascade) | 2059 | thyroiddiagn. (TSH if needed FT4) | SCHK | B | KC | thyroid cascade |
|  | FT4 | 348 | T4 Free thyroxine | FT4 | B | KC | FT4 |
|  | T3 | 347 | T3 Free thyroxine | FT3 | B | KC | T3 |
|  | TSH-receptor stimulating antibodies | 1390 | TSH receptor ab. | TSRI | B | KC | TSI |
|  | thyroid peroxidase (TPO) antibodies | 1365 | thyroidtissue ab. | THYI | B | IM | TPO-Ab |
|  | ESR | 134 | sedimentation (ESR) | BSE | B | HE | ESR |
|  | leucocytes | 547 | leucocytes | LEUK | B | HE | Leuco diff |
|  | leucocytes differentiation | 241 | leucocytes, differentiation | DIFF | B | HE | Leuco diff |
|  | ultrasound thyroidgland | 2415 | ultrasound thyroidgland | ECHO | TA | BO | US thyroid |
|  |  |  |  |  |  |  |  |
| **Urinary tract infections** | Urine culture with antibiotic resistance testing | 1338 | urine culture | STAK | U | BA | urine culture |
|  |  |  |  |  |  |  |  |
| **Dyslipaedemia** | Total cholesterol | 192 | Total cholesterol | CHOL | B | KC | T-cholesterol |
|  | HDL | 446 | HDL-cholesterol | HDL | B | KC | HDL |
|  | LDL | 542 | LDL-cholesterol | LDL | B | KC | LDL |
|  | triglycerides | 1377 | triglyceriden | TRIG | B | KC | triglycerides |
|  | total cholesterol/ HDL ratio | 181 | cholesterol/HDL-cholesterol ratio | CHHD | B | KC | ratio T-chol/HDL |
|  | Homocysteine | 470 | Homocysteine | HOMC | B | KC | Homocysteine |
|  |  |  |  |  |  |  |  |
| **perimenopausal complaints** | follikel stimulating hormone (FSH) | 346 | follikelstimulating hormone | FSH | B | KC | FSH |
|  | luteinising hormoon (LH) | 553 | luteinising hormoon (LH) | LH | B | KC | LH |
|  | estradiol | 768 | estradiol | OEST | B | KC | estradiol |
